# Sexually transmitted infection testing and key outcomes following implementation of online postal self-sampling into sexual health services in England: a retrospective observational study of routinely collected service-level healthcare data

**DOI:** 10.1016/j.lanepe.2025.101541

**Published:** 2025-11-29

**Authors:** Jo Gibbs, Oliver Stirrup, Anna Tostevin, Alison Howarth, Claire Dewsnap, Jonathan D.C. Ross, Andy Williams, Vicky Tittle, Sara Day, Jack Brown, David Crundwell, Louise J. Jackson, Catherine H. Mercer, Jessica Sheringham, Ann Sullivan, Andrew J. Winter, Geoff Wong, Andrew Copas, Fiona Burns

**Affiliations:** a UCL Institute for Global Health, Mortimer Market Centre, Off Capper Street, London, WC1E 6JB, UK; b Sheffield Teaching Hospitals NHS Foundation Trust, Sheffield, UK; c Birmingham City University, Birmingham, UK; d University of Birmingham, Birmingham, UK; e Barts Health NHS Trust, London, UK; f Chelsea and Westminster Hospital NHS Foundation Trust, London, UK; g Lay Representative, London, UK; h Institute of Applied Health Research, University of Birmingham, Birmingham, UK; i Institute of Epidemiology and Health Care, University College London, London, UK; j Sandyford Sexual Health, NHS Greater Glasgow and Clyde, Glasgow, UK; k Nuffield Department of Primary Care Health Sciences, University of Oxford, Oxford, UK

**Keywords:** STIs, Online STI testing, STI service provision, Digital health, Sexual health

## Abstract

**Background:**

A shift to online postal self-sampling (OPSS) for sexually transmitted infections (STIs) in high-income settings has occurred. We evaluate whether introduction of OPSS in England is associated with changes in testing activity and if this differs by population characteristics.

**Methods:**

A retrospective study of sexual health (online and clinic-based) service-level data, across three case study areas (CSAs) that implemented OPSS at different times, using different models, and whose populations have different socio-demographic profiles, between 01/01/2015 and 31/12/2022 (from 01/08/2014 in CSA1 to ensure 12 months pre-OPSS). The primary outcome was chlamydia/gonorrhoea and HIV testing activity. We evaluated change over time using selected calendar years, with total activity following introduction of OPSS (2019 and 2022) compared to pre-OPSS periods (CSA1, 2014–2015, CSA2 2017, CSA3 2019), and whether outcome changes differed by socio-demographic characteristics.

**Findings:**

In all CSAs chlamydia/gonorrhoea and HIV testing activity increased following introduction of OPSS with incidence rate ratios (IRR) for chlamydia/gonorrhoea testing in 2022 compared to pre-OPSS baseline ranging from 2.1 (95% CI 2.1–2.2) in CSA1 to 2.5 (95% CI 2.4–2.5) in CSA3, and for HIV testing from 1.5 (95% CI 1.5–1.5) in CSA1 to 2.8 (95% CI 2.8–2.8) in CSA2. Differences existed across all demographic characteristics in the relative change in testing incidence (all P < 0.0001 for chlamydia/gonorrhoea). Higher testing activity via OPSS was seen among men who have sex with men (MSM), particularly in CSAs1-2 for chlamydia/gonorrhoea (IRR2.9 (95% CI 2.8–3.1) and 3.6 (95% CI 3.5–3.7) in MSM compared to 1.7 (95% CI 1.7–1.8)and 1.8 (95% CI 1.8–1.8) in men who have sex exclusively with women (MSEW) for 2022 *vs* pre-OPSS). In CSA3, the largest relative increase occurred in women (IRR 3.2 (95% CI 3.1–3.3), compared to IRR 1.9 (95% CI 1.8–1.9) in MSEW). The most deprived areas had the lowest relative increase in chlamydia/gonorrhoea testing uptake (1.9–2.1 for CSA1-3).

**Interpretation:**

Despite a reduction in clinic-based testing linked to COVID-19, the introduction of OPSS has been associated with increases in overall testing activity. OPSS uptake was lower among populations with greater potential for unmet need, such as individuals living in more deprived areas. Although OPSS is available to all people living within the commissioned areas, in practice not all individuals with a need for STI testing are aware of it or have the confidence and ability to access it. Differences across all socio-demographic characteristics in the relative change in testing could inadvertently increase existing inequalities in access to care and it is important to offer choice of mode of testing for service users.

**Funding:**

10.13039/501100000272National Institute for Health and Care Research.

**Research in context**

**Evidence before this study**

Building on a scoping review that evaluated the evidence up until July 2021, in March 2025 we searched PubMed and Web of Science without language restrictions for studies on online postal self-sampling (OPSS) and clinic-based sexually transmitted infection (STI) testing published in the past 5 years. Search terms used were “STI” or “Sexually Transmitted Diseases” AND “home-based” OR “self-sampling” OR “online” or “digital” and searched within these articles for those referencing clinic-based services. Whilst digital health interventions have the potential to increase access, there is currently a dearth of knowledge of the impact on clinical and public health outcomes compared to testing in clinic-based settings. There is evidence that OPSS can increase overall testing activity, and that women, those of white ethnicity and living in less deprived areas are more likely to access testing via OPSS. There have been no multi-site evaluations evaluating OPSS and clinic-based sexual health service testing within the UK.

**Added value of this study**

To our knowledge, this is the largest evaluation of the impact of OPSS on STI testing including both online and clinic-based service-level data over time, from 3 large case study areas (CSAs) that implemented OPSS in different ways. We have demonstrated that the introduction of OPSS into the sexual health economy leads to an increase in overall testing activity, even when access to clinic-based services is restricted. The greatest relative increase in testing activity were seen in populations that already had greater testing activity prior to OPSS being implemented. Although OPSS chlamydia and gonorrhoea test positivity was mostly lower than clinic-based tests across time and different gender and sexual behaviour groups, OPSS accounts for the majority of new chlamydia diagnoses in 2022 in two of the CSAs. This study has shown that there are restrictions in the ability to track a user journey across hybrid online and clinic-based settings, particularly where an external OPSS provider was used, with large variations in which and how variables were captured within individual electronic health record systems. This limited the ability to capture key clinical outcomes for all testing activity, and to be able to provide a more granular analysis of population groups.

**Implications of all the available evidence**

We have demonstrated that the introduction of OPSS into sexual health service delivery is associated with an increase in testing activity across population groups. However, the increase varied between groups, with a shift in testing away from clinic-based to online testing in all CSAs, and OPSS uptake was lower among populations at greater risk of unmet need. We show that there is a need to maintain access to clinic-based testing for those individuals and populations who are unable or unwilling to access testing online. Further research is required to improve data quality to monitor outcomes, with a need for standardised and reportable outcomes to be developed for OPSS as well as clinic-based services so that future data can be more comparable across all the services, as well as facilitating data linkage across settings. STI testing only addresses one aspect of sexual health, and further research is required to understand if the changes in sexual health service delivery are impacting other measures of unmet need, for example, primary prevention of STIs and contraception.

## Introduction

Globally, poor sexual health remains a major public health challenge.^1^^,^^2^ Within the UK, the incidence of key sexually transmitted infections (STIs) continues to rise, disproportionately affecting certain groups including young people aged 15–24, some Black ethnic minorities, and men who have sex with men (MSM).^3^

Sexual health service providers have been at the forefront of utilising developments in digital health and diagnostics to widen access to care, address unmet need, and mitigate the challenge of limited resources.^4^, ^5^, ^6^ Sexual health services in England underwent major system change due to the Health and Social Care Act 2012.^7^ As a result of this legislation, care became more fragmented with HIV services, most sexual health services (SHS), and other reproductive services being commissioned by different entities.^7^ It also resulted in the majority of SHS being delivered within a reduced, fixed budget at time of increasing demand^8^—encouraging adoption of innovative service delivery models.^7^

Over the past 10-years, access to screening for key STIs via SHS has been revolutionised by the availability of online postal self-sampling (OPSS). Within England, OPSS services have been implemented at different times using different models, with some added as a bolt-on and integrated with existing services and others delivered by an external provider with no direct integration with existing services.^9^ Service users can access testing for STIs (including chlamydia, gonorrhoea, HIV, and syphilis) online and have a self-sampling kit sent to an address of their choice, or to collect a test kit from a clinic or pharmacy.^10^ Once they have taken the samples then they send the kit through the post to the laboratory. This has changed how services are provided, with some clinic-based services no longer offering in-person screening for those without symptoms. Emerging evidence suggests some groups, particularly racially minoritised groups, and those living in more deprived areas, are less likely to access this testing via OPSS and as such, OPSS has the potential to widen existing inequalities.^10^^,^^11^

An analysis of surveillance data showed that in England, although overall testing activity decreased between 2015 and 2022, there was an increase of 36% in the proportion of chlamydia tests originating from OPSS between 2015 (2%) and 2022 (38%), with groups who access in person compared to online testing differing in some key respects.^12^ However, this analysis was limited by the variables captured, missing data and the inability of routine surveillance systems to link people across episodes of care.

This study was undertaken as part of ASSIST, a mixed-methods realist evaluation of online postal self-sampling.^13^ It aims to establish the change in access to care and service delivery since the introduction of OPSS services, the impact on health inequalities and key clinical and public sexual health outcomes, and to identify who is accessing online services and clinic-based services.

## Methods

### Study design and data sources

We conducted a retrospective observational study of routinely collected service-level healthcare data from users of OPSS and clinic-based SHS in three case study areas (CSA1-3) in England. The three CSAs cover primarily urban and sub-urban areas and were selected to maximise socio-demographic and geographical diversity, and because of the differing timing and models of implementation. OPSS was introduced in August 2015 in CSA1, in January 2018 in CSA2 and in January 2020 in CSA3. The primary study period for the evaluation was 2015–2022, but data from 2014 were also included for CSA1 to provide at least one year of clinic data prior to the introduction of OPSS. For CSA1, OPSS was provided by and fully integrated with the local SHS, whereas for CSA2 and CSA3, OPSS was run by organisations separate from local clinics (with no direct person-level linkage between OPSS and clinic datasets). For CSA2 and CSA3 most people with positive or reactive results were transferred to local clinic services for further care, although postal treatment for chlamydia was available for uncomplicated cases detected through OPSS in CSA2.

Data were included for individuals aged ≥16 years. Due to the size of CSA2, two clinic-based services were included within the study and the same geographic restriction was applied to the OPSS service data to enable reasonable comparison of testing rates over time within the same population. Whilst OPSS services are restricted to those living in the commissioned areas, anyone can access SHS. Geographical inclusion criteria were applied to each CSA based on eligibility for use of the local OPSS service according to postcode and the core catchment areas of clinic services. Key aspects of the three CSAs are summarised in Table 1.

**Table 1:** Characteristics of the three case study areas.

	CSA 1	CSA 2	CSA 3
Urban population size	C1.1 m	C8m	C450,000
Date OPSS implemented	08/2014	02/2018	02/2019
Number of sexual health services	1	2	1
Model & commissioning	Integrated service; local	External provider; consortium	External provider; individual local authority
Pre-OPSS years	Aug 2014–July 2015	2017	2019
Restrictions	None	4 kits/year	Low risk heterosexuals & WSM can only test for HIV/syphilis once a year
Symptomatic users^a^	Sign posted to clinic but able to access	Minimally symptomatic able to access from 2020	Only asymptomatic able to access
Online chlamydia treatment pathway	x	✓	x
HIV Ab/Ag testing based on reported HIV status	x	✓	✓

a CSA 1 do not capture if a service user is symptomatic, and in CSA 2 and 3, as with clinic-based services, it is dependent on service user reporting presence of symptoms.

### Procedures

#### Outcomes

The primary outcome was chlamydia, gonorrhoea and HIV testing activity. Secondary outcomes included rates of new diagnoses of chlamydia, gonorrhoea and HIV, proportion of people diagnosed with chlamydia or gonorrhoea receiving treatment, proportion of people testing positive for gonorrhoea who have a test of cure, proportion of people with reactive tests who have confirmatory HIV test, and time to treatment for chlamydia. The outcomes of ‘partner notification rates for those diagnosed with chlamydia, gonorrhoea and HIV’ were described in the protocol, but were dropped from the statistical analysis plan because of a lack of useable data across the study sites. The study protocol also included evaluation of testing activity, diagnosis rates and confirmatory testing for syphilis, but we plan to report this separately due to the additional complexity of modes of testing for this infection.

#### Definitions and variables

For all infection types, evaluation of primary and secondary outcomes was based on episodes of care with a duration of up to 6 weeks (to avoid double-counting of tests or diagnoses in line with standard practice for STI surveillance).^14^ Episodes of care started at initial appointment date for clinic datasets and online triage date for OPSS datasets, defined separately for each outcome. For OPSS datasets, testing activity was considered to have taken place when a sample was returned for any given individual. For CSA1, each episode of care was assigned as ‘clinic-based’ or ‘OPSS-based’ according to the sample source of the first recorded test.

For both chlamydia and gonorrhoea, tests are nucleic acid amplification tests (NAAT) using urine or swabs at any anatomical location, with at least one positive result within an episode of care counted as a new diagnosis. Successful treatment was defined as an appropriate antibiotic prescription within 1 week prior to or up to 6 weeks after the sample date for the first positive result. For the OPSS datasets in CSA2 and CSA3 transfer of care to a clinic service was regarded as a successful treatment outcome; postal treatment for chlamydia was also available for some cases in CSA2. Test of cure for gonorrhoea was defined as any NAAT test 2–6 weeks after treatment date; this was only evaluated among those testing in clinic, including those who had tested positive via OPSS and had a repeat NAAT taken in clinic, with recorded successful treatment. Time to treatment for chlamydia was evaluated for all cases in CSA1, clinic cases and those OPSS cases obtaining postal treatment following an online clinical consultation in CSA2 and for clinic cases only in CSA3.

HIV testing activity was based on antibody/antigen screening tests with a processable blood sample., with service users using lancets to collect small volumes of blood in a microtainer. Any HIV screening tests conducted on people with known HIV (determined from diagnostic tests, diagnosis codes and/or self-report) were excluded. A screen-positive result was considered a new diagnosis when there was a clinic record code denoting a new HIV diagnosis within a 6-week interval either side of the test result. For OPSS datasets in CSA2 and CSA3, new diagnoses were defined as those confirmed on routine clinical review by service staff. Further details regarding treatment definitions for chlamydia and gonorrhoea, time-to-treatment outcomes for chlamydia and HIV confirmatory testing are given in Supplementary Appendices.

Population size estimates for each CSA, divided by demographic characteristics, were obtained from National Census 2021 data. For outcomes by index of multiple deprivation (IMD), mid-2019 estimates of population size for each lower layer super output area provided by the Office for National Statistics were used. Age was categorised by completed years: 16–24, 25–34, 35–44, 45–54, 55–64, ≥65. Outcomes were evaluated according to the following groups: all women (including trans women), men recorded as men who have sex with men (including trans men and men who have sex with men and women), and men who have sex exclusively with women (MSEW). We assumed that MSM are likely to be recorded as MSM within clinic databases, whilst for some sites, information on sexual behaviour/sexual orientation may be left blank for MSEW. Groups were defined based on sexual behaviour where recorded, but we also used recorded sexual orientation if explicit information on sexual behaviour was not available. Ethnicity was categorised using Census categories^15^ as: White—British/Irish, White—other, any mixed ethnicity, Asian, Black Caribbean or other, Black African, any other. IMD was evaluated using quintile categories.

### Statistical analysis

For each outcome, we evaluated whether there had been change over time (with total, i.e. clinic and OPSS, activity after introduction of OPSS compared to pre-OPSS periods) within each CSA, and whether changes in outcomes differed by population characteristics of age, gender and sexual behaviour, ethnicity, and IMD. Analyses were conducted separately for each CSA.

The study protocol stated that regression models would be used to separate out the effects of long-term time trends vs the introduction of OPSS vs the impact of COVID-19. However, on initial review of the datasets this was determined to be unfeasible due to the complexity of patterns of change over time and substantial differences between sites. Instead, when writing the Statistical Analysis Plan, we selected 1-year periods for comparison before and after the introduction of OPSS and the COVID-19 pandemic at each CSA: pre-OPSS (baseline) years were Aug 2014–July 2015 for CSA1, 2017 for CSA2 and 2019 for CSA3; 2019 was included for CSA1 and CSA2 as the final year of OPSS pre-COVID-19; and all CSAs were evaluated with OPSS running in 2022 (there were no social distancing rules in place in 2022, and the legal requirement for self-isolation of SARS-CoV-2 cases ended in February 2022). Graphical comparisons explore changes in testing outcomes over the full study period.

Incidence outcomes relating to testing activity and new diagnoses were analysed using Poisson regression models for count data, with incidence expressed per 100,000 person-years (100kPY) for HIV diagnoses and per 100 PY for other outcomes. Binary outcomes (e.g., treatment success) were analysed using a log-link generalised linear model (estimating relative risk (RR) values), with cluster-robust standard errors grouped by patient. Time-to-treatment for chlamydia was analysed using linear regression models.

For each outcome, overall and within each level of demographic variables, we produced statistical comparisons of each comparison year to the pre-OPSS baseline year (expressed as incidence rate ratios (IRRs), RRs or mean difference), without adjustment for the other variables. For each comparison year separately, we then calculated a P-value testing the null hypothesis that the IRR, RR or mean difference is identical across all levels of the demographic variable. For testing activity and new diagnoses of each infection, we also conducted a multivariable comparison (expressed as adjusted IRRs (aIRRs)) for outcomes in 2022 compared to baseline to investigate the factors driving changes in incidence rates.

Where a variable had <1% missing data in the dataset, affected individuals were excluded from all analyses. Substantial missing data was only present for ethnicity, and in this case affected individuals were retained; we used person-level multiple imputation of missing ethnicity values and combined the results with the repeat episode of care observations for each person. Ten imputed datasets were created and analysed for each CSA (further details in Supplementary Appendix).

Data processing and statistical analysis was carried out within the UCL Data Safe Haven and conducted using Stata V18.0, with graphs generated using GGplot2 for R V4.3.0.

### Ethical approval

Ethics approval for the ASSIST study was provided by South Central–Berkshire Research Ethics Committee (ref: 21/SC/0223), including to access unconsented de-identified routinely captured data from datasets collected as part of patients’ standard health care.

### Role of the funding source

The funder of the study had no role in study design, data collection, data analysis, data interpretation, or writing of the report.

## Results

The CSAs contained a total population of over 3.4 million people (Table 2). The proportion of people aged 16–34 years was similar across CSAs (35.4%–39.6%), but the proportion of MSM varied from 1.3% (CSA1) to 2.7% (CSA2). There were substantial differences in the ethnicity and deprivation profile of the CSAs, with the proportions of white British people (the largest ethnic group nationally) ranging from 32.2% (CSA2) to 77.8% (CSA3) and of those living in areas below the first quintile of IMD (the most deprived) between 50.3% (CSA1) and 17.9% (CSA2) respectively.

**Table 2:** Demographic characteristics of sexual health service users in each year included in statistical analyses, with reference population data (ONS-2019 for IMD and Census 2021 for other variables) included for comparison.

	CSA1 [Pre-OPSS: Aug 2014–Jul 2015]				CSA2 [Pre-OPSS: 2017]				CSA3 [PreOPSS: 2019]		
	PreOPSS; n (%)	2019; n (%)	2022; n (%)	Census pop.; n (%)	PreOPSS; n (%)	2019; n (%)	2022; n (%)	Census pop.; n (%)	PreOPSS; n (%)	2022; n (%)	Census pop.; n (%)
% OPSS	0%	52.2%	60.1%	NA	0%	41.3%	77.0%	NA	0%	71.6%	NA
Overall	18,139	51,378	36,612	1,064,140	60,981	105,354	114,860	1,880,148	8349	19,082	456,249
Population group											
Women	10,024 (55.3)	31,624 (61.6)	21,650 (59.1)	550,762 (51.8)	34,351 (56.3)	59,108 (56.1)	62,812 (54.7)	970,060 (51.6)	3968 (47.5)	11,102 (58.2)	233,412 (51.2)
MSEW	6640 (36.6)	15,197 (29.6)	11,599 (31.7)	499,294 (46.9)	20,069 (32.9)	31,639 (30.0)	31,663 (27.6)	858,574 (45.7)	3331 (39.9)	5858 (30.7)	215,283 (47.2)
MSM	1475 (8.1)	4557 (8.9)	3363 (9.2)	14,071 (1.3)	6561 (10.8)	14,607 (13.9)	20,385 (17.7)	51,525 (2.7)	1050 (12.6)	2122 (11.1)	7555 (1.7)
Age (years)											
16–24	7329 (40.4)	23,718 (46.2)	14,083 (38.5)	183,631 (17.3)	16,450 (27.0)	29,194 (27.7)	28,467 (24.8)	281,273 (15.0)	3173 (38.0)	9545 (50.0)	82,300 (18.0)
25–34	6848 (37.8)	17,480 (34.0)	13,654 (37.3)	192,447 (18.1)	27,958 (45.8)	50,630 (48.1)	57,160 (49.8)	462,358 (24.6)	3138 (37.6)	6266 (32.8)	79,401 (17.4)
35–44	2533 (14.0)	6575 (12.8)	5904 (16.1)	180,821 (17.0)	10,646 (17.5)	16,942 (16.1)	20,180 (17.6)	384,032 (20.4)	1163 (13.9)	2162 (11.3)	67,176 (14.7)
45–54	1032 (5.7)	2554 (5.0)	2036 (5.6)	167,404 (15.7)	4321 (7.1)	6181 (5.9)	6503 (5.7)	295,633 (15.7)	563 (6.7)	746 (3.9)	68,557 (15.0)
55–64	309 (1.7)	815 (1.6)	730 (2.0)	143,737 (13.5)	1248 (2.0)	1916 (1.8)	2108 (1.8)	218,921 (11.6)	241 (2.9)	289 (1.5)	63,996 (14.0)
≥65	88 (0.5)	236 (0.5)	205 (0.6)	196,087 (18.4)	358 (0.6)	491 (0.5)	442 (0.4)	237,942 (12.7)	71 (0.9)	74 (0.4)	94,820 (20.8)
Ethnicity											
Missing	1325 (7.3)	4680 (9.1)	5861 (16.0)		9684 (15.9)	19,623 (18.6)	6291 (5.5)		491 (5.9)	2332 (12.2)	
White—British/Irish	7075 (39.0)	25,151 (49.0)	15,609 (42.6)	574,827 (54.0)	16,797 (27.5)	34,034 (32.3)	41,816 (36.4)	605,566 (32.2)	5648 (67.6)	12,671 (66.4)	355,143 (77.8)
White—other	1000 (5.5)	2355 (4.6)	1459 (4.0)	44,943 (4.2)	12,793 (21.0)	18,934 (18.0)	23,237 (20.2)	356,187 (18.9)	405 (4.9)	724 (3.8)	18,664 (4.1)
Black African	1265 (7.0)	2808 (5.5)	2235 (6.1)	45,997 (4.3)	4356 (7.1)	6922 (6.6)	9475 (8.2)	129,991 (6.9)	278 (3.3)	613 (3.2)	12,956 (2.8)
Black Caribbean or other	3001 (16.5)	5470 (10.6)	4136 (11.3)	49,857 (4.7)	5161 (8.5)	7211 (6.8)	7955 (6.9)	86,364 (4.6)	227 (2.7)	362 (1.9)	5658 (1.2)
Asian	2518 (13.9)	5720 (11.1)	3645 (10.0)	275,428 (25.9)	6476 (10.6)	8272 (7.9)	9549 (8.3)	495,722 (26.4)	549 (6.6)	780 (4.1)	40,535 (8.9)
Any other	295 (1.6)	769 (1.5)	554 (1.5)	38,892 (3.7)	1475 (2.4)	2889 (2.7)	6426 (5.6)	124,714 (6.6)	140 (1.7)	414 (2.2)	12,232 (2.7)
Mixed ethnicity	1660 (9.2)	4425 (8.6)	3113 (8.5)	34,196 (3.2)	4239 (7.0)	7469 (7.1)	10,111 (8.8)	81,604 (4.3)	611 (7.3)	1186 (6.2)	11,061 (2.4)
IMD											
First quintile (most deprived)	10,463 (57.7)	26,548 (51.7)	18,857 (51.5)	516,875 (48.9)	14,125 (23.2)	22,110 (21.0)	24,458 (21.3)	341,318 (17.6)	2743 (32.9)	5277 (27.7)	154,465 (32.2)
Second quintile	4141 (22.8)	11,399 (22.2)	7931 (21.7)	208,830 (19.8)	27,467 (45.0)	46,486 (44.1)	50,292 (43.8)	758,488 (39.1)	1444 (17.3)	3618 (19.0)	72,043 (15.0)
Third quintile	2335 (12.9)	8058 (15.7)	6104 (16.7)	154,039 (14.6)	11,408 (18.7)	21,361 (20.3)	23,839 (20.8)	446,215 (23.0)	1669 (20.0)	4063 (21.3)	91,563 (19.1)
Fourth quintile	801 (4.4)	2873 (5.6)	2032 (5.6)	81,166 (7.7)	5712 (9.4)	10,990 (10.4)	11,761 (10.2)	265,505 (13.7)	1044 (12.5)	2357 (12.4)	74,551 (15.6)
Fifth quintile (least deprived)	399 (2.2)	2500 (4.9)	1688 (4.6)	95,945 (9.1)	2269 (3.7)	4407 (4.2)	4510 (3.9)	128,432 (6.6)	1449 (17.4)	3767 (19.7)	86,428 (18.0)

Sexual health service users included based on a record of at least one chlamydia test within each year. For CSA2 and CSA3, users of both clinic and OPSS services in any given year will be double-counted as we do not have linkage between datasets.

CSA, case study area; IMD, index of multiple deprivation; MSEW, men who have sex exclusively with women; MSM, men who have sex with men.

**Fig. 1:**
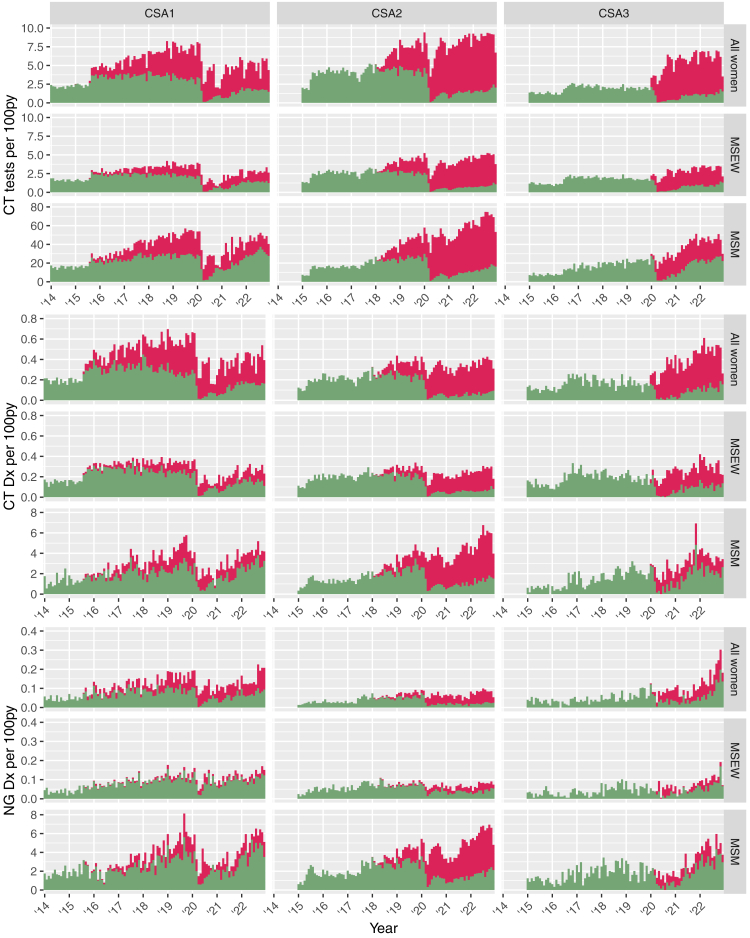
Monthly incidence rates of chlamydia (CT) + gonorrhoea (NG) testing activity and diagnoses of CT and NG. Columns correspond to the three Case Study Areas (CSA1-3), and data for each outcome are disaggregated by sex and sexual behaviour: women, men who have sex exclusively with women (MSEW) and men who have sex with men (MSM). Test performed in clinic are shown in green and online postal self-sampling (OPSS) tests are shown in red.

In all CSAs there was a substantial increase in overall chlamydia testing activity following the introduction of OPSS services, with IRRs for 2022 compared to pre-OPSS baseline ranging from 2.1 (CSA1) to 2.5 (CSA3). Uptake of OPSS testing increased steadily following its introduction in both CSA1 and CSA2, with only a slow corresponding decrease in the level of clinic testing (Fig. 1). There was disruption to clinic services linked to the COVID-19 pandemic (starting March 2020) in all CSAs, which seems to have been particularly associated with the uptake of OPSS testing in CSA2; there has been a gradual recovery in clinic testing throughout 2021 and 2022, but this has not returned to pre-pandemic levels in any of the areas. OPSS was introduced in January 2020 in CSA3, and this timing seems to have led to rapid uptake of OPSS without the gradual change observed in the other CSAs. Identical results were observed for testing activity for chlamydia and gonorrhoea due to use of dual NAAT testing (Table S1).

We detected statistically significant differences across all demographic characteristics in the relative change in chlamydia testing incidence between OPSS periods and pre-OPSS baseline years (Table 3). For example, comparing 2022 to pre-OPSS baseline in CSA1 the level of testing activity was 2.9 times higher in MSM, 2.2 times higher in women and 1.7 times higher in MSEW (P < 0.001 for difference in IRRs across groups); there were also substantial differences in the baseline rates of testing activity between these groups, meaning that the ratio of testing rate in MSM compared to MSEW increased from a 10-fold difference (16 compared to 1.6/100PY) to a 17-fold difference (46 compared to 2.7/100PY). A very similar pattern can be observed in CSA2. In CSA3, the greatest relative increase in testing compared to baseline was observed for women, although MSM again showed the largest absolute increase in incidence rate of testing.

**Table 3:** Statistical evaluation of chlamydia testing activity across analysis years in each case study area (CSA), with full breakdown by demographic characteristics, analysed using Poisson regression models.

	CSA1 [Pre-OPSS: Aug 2014–Jul 2015]				CSA2 [Pre-OPSS: 2017]				CSA3 [PreOPSS: 2019]		
	Pre-OPSS; IR/100PY	Pre-COVID; IR/100PY (IRR comp. Pre-OPSS, 95% CI)	2022; IR/100PY (IRR comp. Pre-OPSS, 95% CI)	2022; aIRR^a^ (95% CI)	Pre-OPSS; IR/100PY	Pre-COVID; IR/100PY (IRR comp. Pre-OPSS, 95% CI)	2022; IR/100PY (IRR comp. Pre-OPSS, 95% CI)	2022; aIRR^a^ (95% CI)	Pre-OPSS; IR/100PY	2022; IR/100PY (IRR comp. Pre-OPSS, 95% CI)	2022; aIRR^a^ (95% CI)
% OPSS	0%	49.8%	56.2%		0%	43.1%	80.6%		0%	72.7%	
Overall	2.1	6.1 (2.9, 2.9–2.9)	4.5 (2.1, 2.1–2.2)	2.3 (2.2–2.4)	3.9	7.2 (1.9, 1.9–1.9)	8.6 (2.2, 2.2–2.2)	2.4 (2.3–2.5)	2.2	5.5 (2.5, 2.4–2.5)	2.3 (2.2–2.4)
Population group *[missing, eq. P]*	0.1%	0.7%, <0.001	0.6%, <0.001		0.1%	0.1%, <0.001	0.0%, <0.001		0.0%	0.0%, <0.001	
Women	2.3	7.3 (3.2, 3.2–3.3)	5.1 (2.2, 2.2–2.3)	Ref.	4.2	7.7 (1.9, 1.8–1.9)	8.8 (2.1, 2.1–2.1)	Ref.	1.9	6.2 (3.2, 3.1–3.3)	Ref.
MSEW	1.6	3.6 (2.3, 2.2–2.3)	2.7 (1.7, 1.7–1.8)	0.8 (0.8–0.8)	2.7	4.4 (1.7, 1.6–1.7)	4.8 (1.8, 1.8–1.8)	0.9 (0.8–0.9)	1.8	3.3 (1.9, 1.8–1.9)	0.6 (0.6–0.6)
MSM	16	52 (3.3, 3.1–3.4)	46 (2.9, 2.8–3.1)	1.3 (1.2–1.4)	19	45 (2.4, 2.4–2.5)	67 (3.6, 3.5–3.7)	1.7 (1.6–1.7)	22	44 (2.0, 1.9–2.1)	0.6 (0.6–0.7)
Age *[missing, eq. P]*	0.0%	0.0%, <0.001	0.0%, <0.001		0.0%	0.0%, <0.001	0.0%, <0.001		0.0%	0.0%, <0.001	
16–24	5.1	16 (3.2, 3.2–3.3)	10 (2.0, 1.9–2.0)	0.9 (0.9–1.0)	7	13 (1.9, 1.9–1.9)	14 (2.0, 2.0–2.0)	0.8 (0.8–0.8)	4.5	15 (3.4, 3.3–3.5)	1.6 (1.5–1.7)
25–34	4.4	12 (2.7, 2.6–2.7)	9.3 (2.1, 2.1–2.2)	Ref.	7.1	14 (2.0, 2.0–2.0)	18 (2.5, 2.4–2.5)	Ref.	4.9	10 (2.1, 2.1–2.2)	Ref.
35–44	1.7	4.5 (2.7, 2.6–2.8)	4.3 (2.6, 2.4–2.7)	1.2 (1.1–1.3)	3.3	5.6 (1.7, 1.7–1.7)	7.3 (2.2, 2.2–2.2)	0.9 (0.9–0.9)	2.1	4.2 (2.0, 1.8–2.1)	0.9 (0.9–1.0)
45–54	0.74	1.9 (2.5, 2.4–2.7)	1.6 (2.2, 2.0–2.3)	1.0 (0.9–1.1)	1.8	2.7 (1.5, 1.5–1.6)	3 (1.7, 1.6–1.8)	0.7 (0.7–0.7)	0.97	1.4 (1.4, 1.3–1.5)	0.7 (0.6–0.7)
55–64	0.26	0.71 (2.7, 2.4–3.0)	0.68 (2.6, 2.3–2.9)	1.2 (1.1–1.4)	0.69	1.1 (1.6, 1.5–1.8)	1.3 (1.9, 1.8–2.0)	0.8 (0.7–0.8)	0.44	0.6 (1.4, 1.2–1.6)	0.6 (0.5–0.7)
≥65	0.05	0.16 (3.0, 2.4–3.7)	0.14 (2.6, 2.1–3.3)	1.2 (1.0–1.5)	0.17	0.26 (1.5, 1.3–1.7)	0.25 (1.5, 1.3–1.6)	0.6 (0.5–0.7)	0.09	0.1 (1.2, 0.9–1.6)	0.6 (0.4–0.7)
Ethnicity *[missing, eq. P]*	7.0%	8.6%, <0.001	14.0%, <0.001		14.5%	17.0%, <0.001	4.5%, <0.001		5.4%	11.0%, <0.001	
White—British/Irish	1.6	5.9 (3.6, 3.6–3.7)	4.1 (2.5, 2.5–2.6)	Ref.	3.9	8.6 (2.2, 2.2–2.2)	10 (2.6, 2.6–2.6)	Ref.	2	5.3 (2.6, 2.6–2.7)	Ref.
White—other	2.9	7 (2.4, 2.2–2.6)	5.3 (1.8, 1.7–1.9)	0.7 (0.7–0.8)	5.1	8.5 (1.7, 1.6–1.7)	9.7 (1.9, 1.9–1.9)	0.7 (0.7–0.7)	2.8	5.6 (2.0, 1.8–2.2)	0.7 (0.7–0.8)
Black African	3.6	8.7 (2.4, 2.3–2.6)	7.8 (2.2, 2.0–2.3)	0.8 (0.8–0.9)	4.8	8.7 (1.8, 1.8–1.9)	11 (2.3, 2.3–2.4)	0.9 (0.9–0.9)	2.7	7 (2.6, 2.3–3.0)	1.0 (0.9–1.1)
Black Carib. or other	8.6	17 (2.0, 1.9–2.0)	14 (1.6, 1.5–1.7)	0.6 (0.6–0.7)	8.7	14 (1.6, 1.5–1.6)	15 (1.7, 1.7–1.8)	0.7 (0.6–0.7)	5.2	9.8 (1.9, 1.6–2.2)	0.7 (0.6–0.8)
Asian	1.2	2.9 (2.5, 2.3–2.6)	2.1 (1.8, 1.7–1.9)	0.7 (0.7–0.8)	1.8	2.7 (1.5, 1.5–1.5)	2.7 (1.5, 1.4–1.5)	0.6 (0.5–0.6)	1.8	3 (1.7, 1.5–1.9)	0.6 (0.6–0.7)
Any other	1	2.8 (2.8, 2.4–3.1)	2.3 (2.3, 2.0–2.6)	0.9 (0.8–1.0)	1.7	3.6 (2.1, 2.0–2.3)	7.4 (4.3, 4.1–4.5)	1.7 (1.6–1.7)	1.7	4.8 (2.9, 2.5–3.5)	1.1 (0.9–1.3)
Mixed ethnicity	6.6	19 (2.9, 2.7–3.1)	14 (2.2, 2.1–2.3)	0.9 (0.8–0.9)	7.6	15 (2.0, 1.9–2.0)	19 (2.5, 2.4–2.6)	1.0 (0.9–1.0)	7.1	16 (2.3, 2.1–2.5)	0.9 (0.8–1.0)
IMD *[missing, eq. P]*	0.0%	0.0%, <0.001	0.0%, <0.001		0.0%	0.0%, <0.001	0.3%, <0.001		0.0%	0.1%, <0.001	
First quintile	2.5	6.6 (2.6, 2.5–2.7)	4.8 (1.9, 1.9–1.9)	Ref.	5	8.4 (1.7, 1.7–1.7)	10 (2.0, 2.0–2.1)	Ref.	2.1	4.5 (2.1, 2.0–2.2)	Ref.
Second quintile	2.5	6.9 (2.8, 2.7–2.9)	5.1 (2.1, 2.0–2.1)	1.1 (1.1–1.1)	4.4	7.9 (1.8, 1.8–1.8)	9.3 (2.2, 2.1–2.2)	1.1 (1.1–1.1)	2.4	6.6 (2.7, 2.6–2.9)	1.3 (1.2–1.4)
Third quintile	1.9	6.5 (3.5, 3.3–3.6)	5.1 (2.7, 2.6–2.9)	1.4 (1.4–1.5)	3	6.2 (2.0, 2.0–2.1)	7.4 (2.5, 2.4–2.5)	1.3 (1.3–1.4)	2.2	5.8 (2.6, 2.5–2.8)	1.3 (1.2–1.4)
Fourth quintile	1.2	4.5 (3.8, 3.5–4.0)	3.3 (2.8, 2.6–3.0)	1.6 (1.5–1.7)	2.5	5.4 (2.1, 2.1–2.2)	6.1 (2.4, 2.4–2.5)	1.4 (1.3–1.4)	1.7	4.1 (2.4, 2.2–2.5)	1.2 (1.1–1.3)
Fifth quintile	0.51	3.3 (6.5, 5.9–7.1)	2.2 (4.3, 3.9–4.7)	2.1 (1.9–2.3)	2	4.4 (2.2, 2.1–2.3)	4.7 (2.3, 2.2–2.4)	1.4 (1.3–1.4)	1.9	5.6 (2.9, 2.7–3.0)	1.7 (1.6–1.8)

Identical results were observed for gonorrhoea testing activity due to the use of dual testing approach throughout the study period.

aIRR, adjusted IRR; eq. P, P-value testing equality of IRR across groups; IR, incidence rate; IRR, incidence rate ratio. IR and IRR values calculated using multiple imputation for ethnicity values, with other missing data excluded.

a With adjustment for all variables listed in table; The ‘overall’ aIRR value represents a comparison to the baseline year for a 25–34 year-old woman of white British/Irish ethnicity living in an area with IMD in the first quintile, for other demographic variables such as age the aIRR values represent how much greater is the change from baseline year in each group of the variable relative to the respective reference group.

Relative changes in the incidence rate of chlamydia testing by age group showed different patterns (Table 3), with lowest increase in uptake in 2022 compared to baseline observed in 16–24-year-olds in CSA1, whereas this was lowest in the oldest age group (≥65) in the other CSAs. The pattern was more consistent for differences by ethnicity, with those who identified as white British/Irish showing the largest or joint largest relative increase in each case (except for ‘any other’ which may be susceptible to data quality issues) and those who identified as Asian showing below average increases from a low baseline level of testing. People living in less deprived areas, as defined by IMD, showed greater relative increases in testing activity, and this trend was particularly clear on adjusted analyses that also accounted for the other demographic characteristics considered.

Trends in the incidence rates of chlamydia diagnoses and the split between cases diagnosed via clinic testing and on OPSS broadly followed in proportion to the pattern observed for testing activity (Fig. 1), with increases from baseline in all cases and most chlamydia diagnoses occurring via OPSS in CSA2 and CSA3 by 2022 (Table 4). There were also substantial increases in incidence rates of gonorrhoea compared to baseline year in all CSAs. However, a relatively low proportion of gonorrhoea diagnoses were made via OPSS in 2022 in both CSA1 (32.8%) and CSA3 (36.2%). Further details with breakdowns by demographic characteristics are given in Supplementary Appendices (Tables S3 and S4), including a full breakdown of available detailed information on sex, gender and sexual behaviour (Table S11). The proportion of chlamydia tests that were positive varied over time, by CSA, and by gender and sexual behaviour (Figure S1). Chlamydia test positivity was higher in clinic-based settings compared to OPSS with the exception of women in CSA2 in 2021 and 2022. Gonorrhoea test positivity per episode of care was also higher in clinic-based settings compared to OPSS (Figure S2). Across all 3 CSAs and over time, MSM had the highest proportion of positive gonorrhoea tests in both clinic-based and OPSS settings.

**Table 4:** Overall summary and results by gender and sexual behaviour grouping for other testing and infection incidence rate outcomes across analysis years in each case study area (CSA), analysed using Poisson regression models.

	CSA1 [Pre-OPSS: Aug 2014–Jul 2015]			CSA2 [Pre-OPSS: 2017]			CSA3 [PreOPSS: 2019]	
	Pre-OPSS; IR/100PY	Pre-COVID; IR/100PY (IRR comp. Pre-OPSS, 95% CI)	2022; IR/100PY (IRR comp. Pre-OPSS, 95% CI)	Pre-OPSS; IR/100PY	Pre-COVID; IR/100PY (IRR comp. Pre-OPSS, 95% CI)	2022; IR/100PY (IRR comp. Pre-OPSS, 95% CI)	Pre-OPSS; IR/100PY	2022; IR/100PY (IRR comp. Pre-OPSS, 95% CI)
**HIV testing activity**								
% OPSS	0%	35.8%	38.3%	0%	44.2%	82.6%	0%	63.3%
Overall	1.9	4.2 (2.2, 2.2–2.2)	2.9 (1.5, 1.5–1.5)	2.4	5.5 (2.3, 2.2–2.3)	6.9 (2.8, 2.8–2.9)	2	3.8 (1.8, 1.8–1.9)
Population group *[missing, eq. P]*	0.1%	0.6%, <0.001	0.6%, <0.001	0.1%	0.1%, <0.001	0.0%, <0.001	0.0%	0.0%, <0.001
Women	2	4.4 (2.2, 2.2–2.3)	2.8 (1.4, 1.4–1.5)	2.4	5.5 (2.3, 2.3–2.3)	6.7 (2.8, 2.8–2.8)	1.8	3.8 (2.1, 2.1–2.2)
MSEW	1.5	2.9 (1.9, 1.8–1.9)	2 (1.3, 1.3–1.3)	1.9	3.7 (1.9, 1.9–1.9)	4 (2.1, 2.1–2.1)	1.7	2.4 (1.5, 1.4–1.5)
MSM	15	44 (3.0, 2.8–3.1)	39 (2.6, 2.5–2.7)	11	36 (3.2, 3.1–3.2)	57 (5.1, 4.9–5.2)	21	40 (2.0, 1.8–2.1)
**Chlamydia diagnoses**								
% OPSS	0%	45.1%	49.6%	0%	31.9%	77.5%	0%	65.7%
Overall	0.18	0.52 (2.8, 2.7–3.0)	0.39 (2.1, 2.0–2.3)	0.25	0.43 (1.7, 1.6–1.7)	0.46 (1.8, 1.7–1.9)	0.2	0.46 (2.2, 2.1–2.4)
Population group *[missing, eq. P]*	0.2%	0.7%, <0.001	0.5%, <0.001	0.3%	0.1%, <0.001	0.0%, <0.001	0.0%	0.0%, <0.001
Women	0.18	0.59 (3.2, 3.0–3.4)	0.42 (2.2, 2.1–2.4)	0.22	0.38 (1.7, 1.6–1.8)	0.37 (1.7, 1.6–1.8)	0.16	0.49 (3.1, 2.8–3.5)
MSEW	0.15	0.34 (2.3, 2.1–2.5)	0.26 (1.7, 1.6–1.9)	0.21	0.29 (1.3, 1.3–1.4)	0.26 (1.2, 1.2–1.3)	0.18	0.31 (1.7, 1.5–1.9)
MSM	1.4	4.4 (3.2, 2.8–3.8)	4.1 (3.0, 2.6–3.6)	1.6	3.8 (2.4, 2.2–2.6)	5.3 (3.4, 3.1–3.6)	2.2	3.6 (1.6, 1.3–2.0)
**Gonorrhoea diagnoses**								
% OPSS	0%	29.7%	32.8%	0%	25.3%	66.7%	0%	36.2%
Overall	0.07	0.21 (3.3, 3.0–3.5)	0.21 (3.2, 3.0–3.5)	0.11	0.19 (1.7, 1.6–1.8)	0.24 (2.2, 2.1–2.3)	0.09	0.21 (2.3, 2.1–2.6)
Population group *[missing, eq. P]*	0.6%	1.3%, 0.084	1.0%, 0.255	0.4%	0.2%, <0.001	0.1%, <0.001	0.0%	0.1%, 0.078
Women	0.04	0.16 (3.6, 3.2–4.2)	0.16 (3.5, 3.0–4.0)	0.04	0.07 (1.8, 1.6–2.0)	0.08 (2.0, 1.8–2.2)	0.06	0.17 (2.7, 2.2–3.2)
MSEW	0.04	0.12 (3.3, 2.8–3.8)	0.13 (3.3, 2.8–3.9)	0.07	0.08 (1.2, 1.0–1.3)	0.07 (1.1, 1.0–1.2)	0.05	0.11 (1.9, 1.5–2.4)
MSM	1.9	5.4 (2.9, 2.5–3.3)	5.5 (3.0, 2.6–3.4)	2.2	4.2 (2.0, 1.8–2.1)	6.1 (2.8, 2.6–3.0)	2	4.5 (2.3, 1.9–2.7)
**HIV diagnoses**		/100kPY	/100kPY	/100kPY	/100kPY	/100kPY	/100kPY	/100kPY
% OPSS	0%	7.1%	11.8%	0%	11.4%	24.0%	0%	38.5%
Overall	4.8	4 (0.8, 0.5–1.2)	1.6 (0.3, 0.2–0.6)	6.2	4.7 (0.8, 0.6–1.0)	2.7 (0.4, 0.3–0.6)	0.66	2.9 (4.3, 1.2–15.2)
Population group *[missing, eq. P]*	0.0%	0.0%, 0.699	0.0%, 0.802	0.8%	2.2%, 0.256	0.0%, 0.273	0.0%	0.0%, 0.856
Women	2.4	1.6 (0.7, 0.3–1.6)	0.54 (0.2, 0.1–0.8)	2.2	2.1 (1.0, 0.5–1.8)	0.82 (0.4, 0.2–0.9)	0	0.86
MSEW	2.6	2.8 (1.1, 0.5–2.3)	1 (0.4, 0.1–1.1)	3.6	1.8 (0.5, 0.3–0.9)	0.93 (0.3, 0.1–0.6)	0.46	0.93 (2.0, 0.2–22.1)
MSM	178	135 (0.8, 0.4–1.4)	64 (0.4, 0.2–0.8)	126	103 (0.8, 0.6–1.2)	66 (0.5, 0.3–0.8)	26	119 (4.5, 1.0–20.8)

**Fig. 2:**
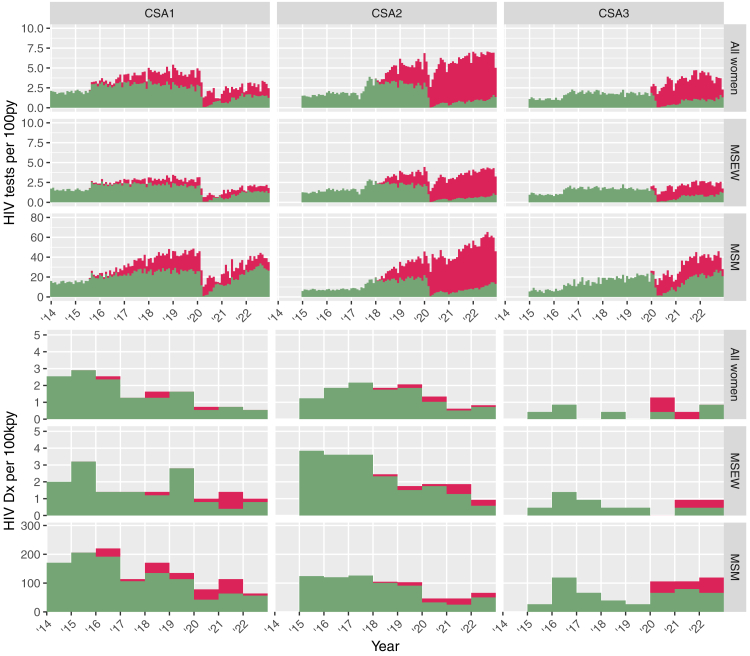
Monthly incidence rates of HIV testing activity and annual incidence rate of HIV diagnoses. Columns correspond to the three Case Study Areas (CSA1-3), and data for each outcome are also disaggregated by sex and sexual behaviour into: women, men who have sex exclusively with women (MSEW) and men who have sex with men (MSM). HIV diagnoses are defined as reactive (or equivocal) antibody tests with an associated new diagnosis code for HIV within 6 weeks, where no indication of a prior HIV diagnosis is recorded. Test performed in clinic are shown in green and online postal self-sampling (OPSS) tests are shown in red.

HIV testing activity showed a similar increase to that for chlamydia testing across the CSAs (Fig. 2, Table 4 and Table S2), albeit with a lower uptake of OPSS—particularly for CSA1, where most HIV tests were still conducted in clinic in 2022. CSA1 and CSA2 showed overall downward trends in the incidence rates of new HIV diagnoses throughout the study period. Fewer than 40% of HIV diagnoses were made based on OPSS testing in any CSA in 2022, despite more tests being completed via OPSS than in clinic for CSA2 and CSA3 (82.6% and 63.3%, respectively) (Table 4 and Table S5).

The introduction of OPSS does not seem to have negatively impacted treatment outcomes for chlamydia and gonorrhoea (Tables S6 and S7), but differences in outcome definitions between OPSS and clinic datasets make detailed comparison difficult. Average time-to treatment (±SD) for chlamydia increased from 4.7 ± 7.5 (preOPSS) to 10.7 ± 8.3 (2022) days in CSA1, but decreased from 5.2 ± 6.6 to 3.6 ± 3.9 days in the equivalent comparison for CSA2 (where, among OPSS users, this could only be measured for those obtaining postal treatment (medication being posted to an individual) via an online clinical consultation) (Table S8); no meaningful comparison is possible for CSA3, where no time-to-treatment results could be obtained for OPSS users.

The proportion of new gonorrhoea diagnoses with confirmed treatment that had a test of cure recorded within 2–6 weeks after treatment date, among those testing in clinics, reduced across all CSAs when comparing pre-OPSS and 2022 (Table S9). The proportion of HIV reactive results who have confirmatory testing recorded within 6 weeks among those with initial testing in clinics, reduced from 40% pre-OPSS to 38.5% in 2022 in CSA1, but rose from 77.2% to 78.5% in CSA2, and from 20% to 34.6% in CSA3 (see Table S10).

## Discussion

This is the largest evaluation of the impact of OPSS on STI testing, diagnosis and treatment outcomes in the UK using clinic-based and online service-level healthcare data from service providers. We have demonstrated that the introduction of OPSS into sexual health service delivery is associated with an increase in testing activity across population groups, but the increase varied between groups, with a shift in testing away from clinic-based to online testing in all CSAs. The impact of OPSS on testing varied by CSA; In CSA2 and 3, where OPSS was provided by an external provider, OPSS uptake and the shift away from clinic-based testing was accelerated by the COVID-19 pandemic and in these areas we observed an overall increase in testing for chlamydia and gonorrhoea. In CSA1, where OPSS was provided in-house, the greatest uptake of OPSS testing for chlamydia and gonorrhoea was seen prior to the COVID-19 pandemic and in 2022 testing dropped compared to pre-pandemic. Similar patterns were found for HIV testing, except the uptake of HIV testing via OPSS was lower than for chlamydia and gonorrhoea testing, reflecting the lower return rates for blood samples and the lower proportion of processable samples compared to NAAT testing.^16^ These findings need to be interpreted in the context of the disruption caused by the COVID-19 pandemic; in CSA1, competing priorities meant that resources were diverted away from the OPSS service.^9^ Whereas in CSA2 and 3 the OPSS provider was able to scale-up their provision to compensate for the reduction in clinic-based services.^9^

The shift from clinic-based to predominantly OPSS testing is associated with differential change according to population characteristics and across CSAs. It has further increased the overall higher rates of chlamydia and HIV testing among MSM, with the lowest uptake seen among MSEW. There were differences in the relative changes in chlamydia testing incidence rate by gender and sexual behaviour, and age group between the different CSAs. Across CSAs and infections, MSEW appear to gain the least benefit from the change in testing pathways, with HIV testing across services (OPSS and clinic-based) remaining relatively static in CSA2 and 3 but declining in CSA1 (decline seen only between pre-COVID and 2022 in CSA1).

More consistent differences were seen by ethnicity and IMD, with those of white British/Irish ethnicity and those living in less deprived areas having the largest or joint largest relative increase in each case study area.

The role of screening for chlamydia and gonorrhoea in high income settings, and who should be targeted for this intervention, is being increasingly questioned.^17^ There is growing evidence that regular screening for STIs in certain key populations including MSM does not lead to change in overall STI prevalence alongside concerns that it could be driving antimicrobial resistance.^17^ The National Chlamydia Screening Programme in England changed their screening policy in 2021 to focus on reducing the reproductive harm of chlamydia, and to therefore only recommend screening women aged 15–24, but men would only be offered testing if an indication had been identified.^18^ This decision was based on the lack of compelling evidence of benefit of screening for chlamydia in young men, who are proportionately much less likely to develop complications compared to women (2% compared to 10–17% respectively).^18^ The findings from this study of an overall increase in testing activity, particularly in MSM, need to be considered within this context.

Test positivity was consistently lower via OPSS testing compared to clinic-based testing. However, proportion of diagnoses made online compared to in clinic was higher in 2022 than pre-COVID for chlamydia, gonorrhoea, and HIV in all CSAs, which likely reflects increased testing activity rather than an increase in underlying incidence. Lower gonorrhoea and chlamydia positivity in those testing online is not unexpected as someone with symptomatic infection may not be eligible for OPSS and/or prefer to access in person testing.^19^

Whilst this analysis provides important new insights, some findings are broadly consistent with what is already known. Analyses of national-level data have demonstrated that the proportion of chlamydia testing occurring within clinic-based settings has decreased between 2015 (43.8%) in 2015–2022 (29.9%), and there is lower chlamydia test positivity in testing that occurs via OPSS compared to clinic-based testing.^12^ Previous observational studies of service level data in single sites within England have found that OPSS users are more likely to be women, of white ethnicity and from less deprived backgrounds^10^^,^^16^^,^^20^^,^^21^ with lower test positivity compared to clinic-based testing.^16^^,^^20^^,^^21^ Internationally, there are a dearth of studies comparing in person and remote self-sampling for STIs, and limited evidence on the impact of OPSS on equity of access to STI testing.^11^ However, a national evaluation of publicly funded chlamydia testing in Sweden between 2013 and 2017, comparing outcomes of those tested online to clinic-based testing, found that usage of online testing gradually increased over time with no decrease seen in clinic-based testing.^22^ This study found similar chlamydia test positivity in those testing online and in person, with those accessing testing in both settings having the highest proportion of chlamydia-positive tests.^22^

There is limited and mixed evidence in terms of linkage to care and treatment outcomes for those with a positive or reactive test via OPSS.^10^ A large urban OPSS service found that 96.7% of people testing positive for chlamydia received treatment either via their online treatment pathway (56.9%), or through another healthcare service (39.7%).^16^ Based on the data available, we found that OPSS does not seem to have led to an overall deterioration in treatment outcomes for chlamydia and gonorrhoea, although there was a significant increase in the average time-to-treatment for chlamydia observed in CSA 1. The proportion of people treated for gonorrhoea in clinic recorded as having a test of cure (in clinic) dropped over time across all CSAs. In CSA2 and 3, this could be partly due to people being signposted to OPSS for their test of cure coupled with the lack of linkage between OPSS and clinic-based datasets.

There are several limitations with this analysis. The population estimates are taken from a single UK census, so we have not adjusted for the changing population composition during the period of this study. Due to the need to maintain confidentiality for individuals accessing sexual health services, with the legal right for SHS to provide care to people anonymously,^23^ an essential component of sexual health services that must be maintained, there is no direct data linkage between clinic and OPSS data for CSA2 and 3. This lack of direct linkage is exacerbated by inadequate data capture regarding referrals from OPSS to clinics within the clinic-based service records. The lack of direct linkage may have led to some double counting of testing activity and diagnoses for individuals using both clinic and OPSS service routes in CSA2 and 3. Quality of data captured, and variables used to capture data varied by OPSS and clinic-based service, which led to the need to collapse some categories into one variable (e.g., sex, gender and sexual behaviour). Issues with data linkage and quality led to incomplete ascertainment of data for several of the secondary outcomes, in particular partner notification (to the extent this could not be included in the analyses) and treatment outcomes. Whilst primarily targeted at asymptomatic users we know that people with symptoms can and do use OPSS services,^20^ with eligibility varying by service, and data on presence of symptoms is not reliably captured across clinic-based and OPSS services. There were differences in who was tested for HIV via OPSS, with all OPSS blood samples tested for HIV in CSA1 whereas people who report living with HIV are not HIV tested in CSA2 and CSA3. Finally, we have not adjusted for the impact of HIV pre-exposure prophylaxis (PrEP) access requirements. People accessing PrEP are required to test for HIV and other STIs on a 3-monthly basis^24^; this is likely to account for some of the uplift seen in testing by MSM.^25^

The introduction of OPSS as an open access service, although limited to commissioned area and by frequency of testing, has been coupled with restrictions on accessing face-to-face clinic-based services which have been primarily financially driven. These concurrent factors have shifted testing to predominantly via OPSS. Given that there has been a differential change in testing activity according to population characteristics and across CSAs, there are concerns that this is potentially leading to widening health inequalities. Maintaining multimodal access in a locality is important, particularly in a system that has a fixed budget, where increasing capacity in OPSS and decreasing capacity in clinic-based settings could result in restricted access to services as a whole.

The difficulty in tracking a person across a hybrid online and clinic-based user journey was exacerbated by using an external OPSS provider.

In conclusion, this is the first study to analyse service level data from both OPSS and clinic-based services, in multiple case study areas, over time and adds important findings in terms of clinical and public outcomes beyond testing activity. When interpreted in combination with evidence on the implementation of OPSS,^9^ the analyses from this paper indicate that the introduction of OPSS is associated with an increase in overall testing activity, although the extent of the increase varies by population group and could lead to widening of health inequalities in access to care if other modes of access are not offered. It is therefore important to provide service users with a choice of ways to test and, as recommended by the World Health Organisation, for online services to complement clinic-based services.^26^ This study has provided important findings to inform the current dearth of evidence around effectiveness of digital health interventions for preventing and managing STIs in diverse populations.^26^

Further research is required to improve data quality to monitor care/outcomes, with a need for standardised and reportable outcomes to be developed for OPSS as well as clinic-based services so that future data can be more comparable across all the services, as well as facilitating data linkage across SHS settings without using a common identifier used in other healthcare settings (e.g., the NHS number). The latter should involve community and other key stakeholders’ opinions. The final year of this study was conducted at a time that services in England were exiting the recovery phase after the COVID-19 pandemic, and further studies are required to understand the longer-term impact of the changes seen in service delivery. Finally, STI testing only addresses one aspect of sexual health, and further research is required to understand if the changes in SHS delivery are impacting other measures of unmet need, for example, primary prevention of STIs and contraception.

## Contributors

The study was conceived by JG and FB (co-principal investigators) and designed by JG, OS, AH, AC and FB. Data management was conducted by OS, AT and AH. OS, AT and AH have directly accessed and verified the underlying data. Data were analysed by OS, with input from JG, OS, AH, AT, AC and FB. The manuscript was drafted by JG and OS. All authors contributed to the interpretation of findings and critically reviewed the manuscript. All authors have read and approved the final version and take responsibility for the decision to submit the manuscript.

## Data sharing statement

The datasets analysed for this observational study are not publicly available due to the sensitive data captured within them and the ethical approval in place for this study. Queries can be directed to the corresponding author.

## Declaration of interests

The following authors declare competing interests:

AC, FB, JG, AH, JR, JS, OS and AWin have received funding from the NIHR. VT has previously received funding from the City of London to conduct a separate evaluation. FB has received grant funding from Gilead Sciences, Viiv Healthcare and MSD. The other authors declare no potential conflicts of interest with respect to the research, authorship, and/or publication of this article.

## Appendix A Supplementary data

Supplementary Figures and Tables

Protocal
