# Supplementary material for: Sexually transmitted infection testing and key outcomes following implementation of online postal self-sampling into sexual health services in England: a retrospective observational study of routinely collected service-level healthcare data
Source: Lancet Reg Health Eur. 2025 Nov 29;61:101541. doi: 10.1016/j.lanepe.2025.101541 (PMC12704373; doi:10.1016/j.lanepe.2025.101541)
Supplement: Supplementary Figures and Tables [file mmc1.docx]

# Supplementary Tables and graphs:

**Table S1:** Gonorrhoea testing activity analysed using Poisson regression models

|  | CSA1 [Pre-OPSS: Aug 2014-Jul 2015] | | | | CSA2 [Pre-OPSS: 2017] | | | | CSA3 [PreOPSS: 2019] | | |
| --- | --- | --- | --- | --- | --- | --- | --- | --- | --- | --- | --- |
|  | **Pre-OPSS;** IR/100PY | **Pre-COVID;** IR/100PY (IRR vs col1, 95%CI) | **2022;** IR/100PY (IRR vs Pre-OPSS, 95%CI) | **2022**; aIRR* (95%CI) | **Pre-OPSS;** IR/100PY | **Pre-COVID;** IR/100PY (IRR vs col1, 95%CI) | **2022;** IR/100PY (IRR vs Pre-OPSS, 95%CI) | **2022**; aIRR (95%CI) | **Pre-OPSS;** IR/100PY | **2022;** IR/100PY (IRR vs Pre-OPSS, 95%CI) | **2022**; aIRR* (95%CI) |
| % OPSS | 0% | 49.8% | 56.2% |  | 0% | 43.1% | 80.6% |  | 0% | 72.4% |  |
| Overall | 2.1 | 6.1 (2.9, 2.9-2.9) | 4.5 (2.1, 2.1-2.2) | 2.3 (2.2-2.4) | 3.9 | 7.2 (1.9, 1.9-1.9) | 8.6 (2.2, 2.2-2.2) | 2.4 (2.3-2.5) | 2.2 | 5.5 (2.5, 2.4-2.6) | 2.3 (2.2-2.4) |
| Population grouping *[missing, eq. P]* | 0.1% | 0.7%, <0.001 | 0.6%, <0.001 |  | 0.1% | 0.1%, <0.001 | 0.0%, <0.001 |  | 0.0% | 0.0%, <0.001 |  |
| Women | 2.3 | 7.3 (3.2, 3.2-3.3) | 5.1 (2.2, 2.2-2.3) | Ref. | 4.2 | 7.7 (1.9, 1.8-1.9) | 8.8 (2.1, 2.1-2.1) | Ref. | 1.9 | 6.2 (3.2, 3.1-3.3) | Ref. |
| MSEW | 1.6 | 3.6 (2.3, 2.2-2.3) | 2.7 (1.7, 1.7-1.8) | 0.8 (0.8-0.8) | 2.7 | 4.4 (1.7, 1.6-1.7) | 4.8 (1.8, 1.8-1.8) | 0.9 (0.8-0.9) | 1.8 | 3.3 (1.9, 1.8-1.9) | 0.6 (0.6-0.6) |
| MSM | 16 | 52 (3.3, 3.1-3.4) | 46 (2.9, 2.8-3.1) | 1.3 (1.2-1.4) | 19 | 45 (2.4, 2.4-2.5) | 67 (3.6, 3.5-3.7) | 1.7 (1.6-1.7) | 22 | 45 (2.0, 1.9-2.1) | 0.6 (0.6-0.7) |
| Age *[missing, eq. P]* | 0.0% | 0.0%, <0.001 | 0.0%, <0.001 |  | 0.0% | 0.0%, <0.001 | 0.0%, <0.001 |  | 0.0% | 0.0%, <0.001 |  |
| 16-24 | 5.1 | 16 (3.2, 3.2-3.3) | 10 (2.0, 1.9-2.0) | 0.9 (0.9-1.0) | 7 | 13 (1.9, 1.9-1.9) | 14 (2.0, 2.0-2.0) | 0.8 (0.8-0.8) | 4.5 | 15 (3.4, 3.3-3.5) | 1.6 (1.5-1.7) |
| 25-34 | 4.4 | 12 (2.7, 2.6-2.7) | 9.3 (2.1, 2.1-2.2) | Ref. | 7.1 | 14 (2.0, 2.0-2.0) | 18 (2.5, 2.4-2.5) | Ref. | 4.9 | 10 (2.1, 2.1-2.2) | Ref. |
| 35-44 | 1.7 | 4.5 (2.7, 2.6-2.8) | 4.3 (2.6, 2.4-2.7) | 1.2 (1.1-1.3) | 3.3 | 5.6 (1.7, 1.7-1.7) | 7.3 (2.2, 2.2-2.2) | 0.9 (0.9-0.9) | 2.1 | 4.2 (2.0, 1.8-2.1) | 0.9 (0.9-1.0) |
| 45-54 | .74 | 1.9 (2.5, 2.4-2.7) | 1.6 (2.2, 2.0-2.3) | 1.0 (0.9-1.1) | 1.8 | 2.7 (1.5, 1.5-1.6) | 3 (1.7, 1.6-1.8) | 0.7 (0.7-0.7) | .97 | 1.4 (1.4, 1.3-1.6) | 0.7 (0.6-0.7) |
| 55-64 | .26 | .71 (2.7, 2.4-3.1) | .68 (2.6, 2.3-2.9) | 1.2 (1.1-1.4) | .69 | 1.1 (1.6, 1.5-1.8) | 1.3 (1.9, 1.8-2.0) | 0.8 (0.7-0.8) | .44 | .6 (1.4, 1.2-1.6) | 0.6 (0.5-0.7) |
| ≥65 | .05 | .16 (3.0, 2.4-3.7) | .14 (2.6, 2.1-3.3) | 1.2 (1.0-1.5) | .17 | .26 (1.5, 1.3-1.7) | .25 (1.5, 1.3-1.6) | 0.6 (0.5-0.7) | .09 | .1 (1.2, 0.9-1.6) | 0.6 (0.4-0.8) |
| Ethnicity *[missing, eq. P]* | 7.0% | 8.6%, <0.001 | 14.0%, <0.001 |  | 14.5% | 17.0%, <0.001 | 4.5%, <0.001 |  | 5.4% | 11.1%, <0.001 |  |
| White – British/Irish | 1.6 | 5.9 (3.6, 3.6-3.7) | 4.1 (2.5, 2.5-2.6) | Ref. | 3.9 | 8.6 (2.2, 2.2-2.2) | 10 (2.6, 2.6-2.6) | Ref. | 2 | 5.3 (2.6, 2.6-2.7) | Ref. |
| White – other | 2.9 | 7 (2.4, 2.2-2.6) | 5.3 (1.8, 1.7-1.9) | 0.7 (0.7-0.8) | 5.1 | 8.5 (1.7, 1.6-1.7) | 9.7 (1.9, 1.9-1.9) | 0.7 (0.7-0.7) | 2.8 | 5.6 (2.0, 1.8-2.2) | 0.7 (0.7-0.8) |
| Black African | 3.6 | 8.7 (2.4, 2.3-2.6) | 7.8 (2.1, 2.0-2.3) | 0.9 (0.8-0.9) | 4.8 | 8.7 (1.8, 1.8-1.9) | 11 (2.3, 2.3-2.4) | 0.9 (0.9-0.9) | 2.7 | 7 (2.6, 2.3-3.0) | 1.0 (0.9-1.1) |
| Black Carib. or other | 8.6 | 17 (2.0, 1.9-2.0) | 14 (1.6, 1.5-1.7) | 0.6 (0.6-0.7) | 8.7 | 14 (1.6, 1.5-1.6) | 15 (1.7, 1.7-1.8) | 0.7 (0.6-0.7) | 5.2 | 9.8 (1.9, 1.6-2.2) | 0.7 (0.6-0.8) |
| Asian | 1.2 | 2.9 (2.5, 2.3-2.6) | 2.1 (1.8, 1.7-1.9) | 0.7 (0.7-0.8) | 1.8 | 2.7 (1.5, 1.5-1.5) | 2.7 (1.5, 1.4-1.5) | 0.6 (0.5-0.6) | 1.8 | 3 (1.7, 1.5-1.9) | 0.6 (0.6-0.7) |
| Any other | 1 | 2.8 (2.8, 2.4-3.1) | 2.3 (2.3, 2.0-2.6) | 0.9 (0.8-1.0) | 1.7 | 3.6 (2.1, 2.0-2.3) | 7.4 (4.3, 4.1-4.5) | 1.7 (1.6-1.7) | 1.7 | 4.8 (2.9, 2.5-3.5) | 1.1 (0.9-1.3) |
| Mixed ethnicity | 6.6 | 19 (2.9, 2.7-3.1) | 14 (2.2, 2.1-2.3) | 0.9 (0.8-0.9) | 7.6 | 15 (2.0, 1.9-2.0) | 19 (2.5, 2.4-2.6) | 1.0 (0.9-1.0) | 7.1 | 16 (2.3, 2.1-2.5) | 0.9 (0.8-1.0) |
| IMD *[missing, eq. P]* | 0.0% | 0.0%, <0.001 | 0.0%, <0.001 |  | 0.0% | 0.0%, <0.001 | 0.3%, <0.001 |  | 0.0% | 0.1%, <0.001 |  |
| First quintile | 2.5 | 6.6 (2.6, 2.6-2.7) | 4.8 (1.9, 1.9-1.9) | Ref. | 5 | 8.4 (1.7, 1.7-1.7) | 10 (2.0, 2.0-2.1) | Ref. | 2.1 | 4.5 (2.1, 2.0-2.2) | Ref. |
| Second quintile | 2.5 | 6.9 (2.8, 2.7-2.9) | 5.1 (2.1, 2.0-2.1) | 1.1 (1.1-1.1) | 4.4 | 7.9 (1.8, 1.8-1.8) | 9.3 (2.2, 2.1-2.2) | 1.1 (1.1-1.1) | 2.4 | 6.7 (2.7, 2.6-2.9) | 1.3 (1.2-1.4) |
| Third quintile | 1.9 | 6.5 (3.5, 3.3-3.6) | 5.1 (2.7, 2.6-2.9) | 1.4 (1.4-1.5) | 3 | 6.2 (2.0, 2.0-2.1) | 7.4 (2.5, 2.4-2.5) | 1.3 (1.3-1.4) | 2.2 | 5.8 (2.6, 2.5-2.8) | 1.3 (1.2-1.4) |
| Fourth quintile | 1.2 | 4.5 (3.8, 3.5-4.0) | 3.3 (2.8, 2.6-3.0) | 1.6 (1.5-1.7) | 2.5 | 5.4 (2.1, 2.1-2.2) | 6.1 (2.4, 2.4-2.5) | 1.4 (1.3-1.4) | 1.7 | 4.1 (2.4, 2.2-2.6) | 1.2 (1.1-1.3) |
| Fifth quintile | .51 | 3.3 (6.5, 5.9-7.1) | 2.2 (4.3, 3.9-4.7) | 2.1 (1.9-2.3) | 2 | 4.4 (2.2, 2.1-2.3) | 4.7 (2.3, 2.2-2.4) | 1.4 (1.3-1.4) | 1.9 | 5.6 (2.9, 2.7-3.0) | 1.7 (1.6-1.8) |

aIRR, adjusted IRR; eq. P, P-value testing equality of IRR across groups; IR, incidence rate; IRR, incidence rate ratio. IR and IRR values calculated using multiple imputation for ethnicity values, with other missing data excluded. With adjustment for all variables listed in table; The ‘overall’ aIRR value represents a comparison to the baseline year for a 25-34 year-old woman of white British/Irish ethnicity living in an area with IMD in the first quintile, for other demographic variables such as age the aIRR values represent how much greater is the change from baseline year in each group of the variable relative to the respective reference group.

**Table S2:** HIV testing activity analysed using Poisson regression models

|  | CSA1 [Pre-OPSS: Aug 2014-Jul 2015] | | | | CSA2 [Pre-OPSS: 2017] | | | | CSA3 [PreOPSS: 2019] | | |
| --- | --- | --- | --- | --- | --- | --- | --- | --- | --- | --- | --- |
|  | **Pre-OPSS;** IR/100PY | **Pre-COVID;** IR/100PY (IRR vs col1, 95%CI) | **2022;** IR/100PY (IRR vs Pre-OPSS, 95%CI) | **2022**; aIRR* (95%CI) | **Pre-OPSS;** IR/100PY | **Pre-COVID;** IR/100PY (IRR vs col1, 95%CI) | **2022;** IR/100PY (IRR vs Pre-OPSS, 95%CI) | **2022**; aIRR (95%CI) | **Pre-OPSS;** IR/100PY | **2022;** IR/100PY (IRR vs Pre-OPSS, 95%CI) | **2022**; aIRR* (95%CI) |
| % OPSS | 0% | 35.8% | 38.3% |  | 0% | 44.2% | 82.6% |  | 0% | 63.3% |  |
| Overall | 1.9 | 4.2 (2.2, 2.2-2.2) | 2.9 (1.5, 1.5-1.5) | 1.4 (1.3-1.4) | 2.4 | 5.5 (2.3, 2.2-2.3) | 6.9 (2.8, 2.8-2.9) | 3.2 (3.1-3.3) | 2 | 3.8 (1.8, 1.8-1.9) | 1.5 (1.4-1.6) |
| Population grouping *[missing, eq. P]* | 0.1% | 0.6%, <0.001 | 0.6%, <0.001 |  | 0.1% | 0.1%, <0.001 | 0.0%, <0.001 |  | 0.0% | 0.0%, <0.001 |  |
| Women | 2 | 4.4 (2.2, 2.2-2.3) | 2.8 (1.4, 1.4-1.5) | Ref. | 2.4 | 5.5 (2.3, 2.3-2.3) | 6.7 (2.8, 2.8-2.8) | Ref. | 1.8 | 3.8 (2.1, 2.1-2.2) | Ref. |
| MSEW | 1.5 | 2.9 (1.9, 1.8-1.9) | 2 (1.3, 1.3-1.3) | 0.9 (0.9-0.9) | 1.9 | 3.7 (1.9, 1.9-1.9) | 4 (2.1, 2.1-2.1) | 0.8 (0.7-0.8) | 1.7 | 2.4 (1.5, 1.4-1.5) | 0.7 (0.6-0.7) |
| MSM | 15 | 44 (3.0, 2.8-3.1) | 39 (2.6, 2.5-2.7) | 1.8 (1.7-1.9) | 11 | 36 (3.2, 3.1-3.2) | 57 (5.1, 4.9-5.2) | 1.8 (1.7-1.8) | 21 | 40 (2.0, 1.8-2.1) | 0.9 (0.8-1.0) |
| Age *[missing, eq. P]* | 0.0% | 0.0%, <0.001 | 0.0%, <0.001 |  | 0.0% | 0.0%, <0.001 | 0.0%, <0.001 |  | 0.0% | 0.0%, <0.001 |  |
| 16-24 | 4.6 | 10 (2.2, 2.2-2.3) | 5.8 (1.3, 1.2-1.3) | 0.8 (0.8-0.9) | 4.4 | 9.8 (2.2, 2.2-2.3) | 11 (2.4, 2.4-2.5) | 0.8 (0.8-0.8) | 4.1 | 9.8 (2.4, 2.3-2.5) | 1.5 (1.4-1.6) |
| 25-34 | 4.1 | 8.5 (2.1, 2.0-2.2) | 6.1 (1.5, 1.5-1.5) | Ref. | 4.6 | 11 (2.4, 2.4-2.5) | 14 (3.1, 3.1-3.2) | Ref. | 4.6 | 7.3 (1.6, 1.5-1.7) | Ref. |
| 35-44 | 1.6 | 3.5 (2.3, 2.2-2.4) | 3.1 (2.0, 1.9-2.1) | 1.3 (1.3-1.4) | 2 | 4.4 (2.1, 2.1-2.2) | 6 (2.9, 2.8-3.0) | 0.9 (0.9-1.0) | 2 | 3.2 (1.6, 1.5-1.8) | 1.0 (1.0-1.1) |
| 45-54 | .7 | 1.5 (2.2, 2.1-2.4) | 1.2 (1.7, 1.6-1.9) | 1.2 (1.1-1.3) | .99 | 1.9 (1.9, 1.8-2.0) | 2.3 (2.3, 2.2-2.4) | 0.7 (0.7-0.8) | .89 | 1.1 (1.2, 1.1-1.4) | 0.8 (0.7-0.9) |
| 55-64 | .25 | .63 (2.5, 2.2-2.9) | .54 (2.2, 1.9-2.4) | 1.4 (1.3-1.6) | .38 | .74 (1.9, 1.8-2.1) | .89 (2.3, 2.2-2.5) | 0.7 (0.7-0.8) | .42 | .49 (1.2, 1.0-1.4) | 0.7 (0.6-0.9) |
| ≥65 | .06 | .15 (2.6, 2.1-3.3) | .13 (2.3, 1.8-2.9) | 1.5 (1.2-1.9) | .11 | .19 (1.7, 1.5-2.0) | .18 (1.7, 1.4-2.0) | 0.5 (0.5-0.6) | .08 | .09 (1.1, 0.8-1.5) | 0.7 (0.5-0.9) |
| Ethnicity *[missing, eq. P]* | 6.6% | 9.0%, <0.001 | 15.4%, <0.001 |  | 14.9% | 17.3%, <0.001 | 4.1%, <0.001 |  | 5.5% | 14.8%, <0.001 |  |
| White – British/Irish | 1.5 | 3.8 (2.5, 2.5-2.6) | 2.4 (1.6, 1.6-1.6) | Ref. | 2.3 | 6.5 (2.8, 2.7-2.8) | 8 (3.4, 3.4-3.5) | Ref. | 1.9 | 3.6 (1.9, 1.9-2.0) | Ref. |
| White – other | 2.7 | 5.4 (2.0, 1.9-2.2) | 3.9 (1.4, 1.3-1.5) | 0.9 (0.8-1.0) | 3.2 | 6.8 (2.1, 2.1-2.2) | 8.3 (2.6, 2.5-2.6) | 0.8 (0.7-0.8) | 2.7 | 4.5 (1.6, 1.5-1.8) | 0.9 (0.8-1.0) |
| Black African | 3.4 | 6.7 (2.0, 1.8-2.1) | 5.7 (1.7, 1.5-1.8) | 1.0 (1.0-1.1) | 3.1 | 6.3 (2.1, 2.0-2.1) | 8.5 (2.8, 2.7-2.9) | 0.8 (0.8-0.8) | 2.5 | 5 (2.0, 1.7-2.3) | 1.0 (0.9-1.2) |
| Black Carib. or other | 7.6 | 12 (1.6, 1.6-1.7) | 9.2 (1.2, 1.2-1.3) | 0.8 (0.7-0.8) | 5.5 | 9.8 (1.8, 1.7-1.8) | 11 (1.9, 1.8-2.0) | 0.6 (0.5-0.6) | 4.8 | 5.9 (1.2, 1.0-1.4) | 0.6 (0.5-0.7) |
| Asian | 1.1 | 2.3 (2.1, 2.0-2.2) | 1.6 (1.5, 1.4-1.5) | 0.9 (0.9-1.0) | 1.2 | 2.1 (1.7, 1.6-1.7) | 2.2 (1.8, 1.7-1.8) | 0.5 (0.5-0.5) | 1.7 | 2.5 (1.4, 1.3-1.6) | 0.7 (0.7-0.8) |
| Any other | .94 | 2.1 (2.3, 2.0-2.6) | 1.7 (1.8, 1.6-2.1) | 1.1 (1.0-1.3) | 1.1 | 2.9 (2.7, 2.6-2.9) | 6.4 (6.1, 5.7-6.5) | 1.8 (1.7-1.9) | 1.6 | 3.9 (2.5, 2.1-3.1) | 1.3 (1.1-1.6) |
| Mixed ethnicity | 5.9 | 13 (2.2, 2.0-2.3) | 9 (1.5, 1.4-1.6) | 0.9 (0.9-1.0) | 4.6 | 11 (2.4, 2.3-2.5) | 15 (3.1, 3.0-3.3) | 0.9 (0.9-1.0) | 6.5 | 11 (1.7, 1.5-1.9) | 0.9 (0.8-1.0) |
| IMD *[missing, eq. P]* | 0.0% | 0.0%, <0.001 | 0.0%, <0.001 |  | 0.0% | 0.1%, <0.001 | 0.4%, <0.001 |  | 0.0% | 0.1%, <0.001 |  |
| First quintile | 2.3 | 4.6 (2.0, 2.0-2.1) | 3.2 (1.4, 1.4-1.4) | Ref. | 3.2 | 6.3 (2.0, 1.9-2.0) | 8 (2.5, 2.5-2.6) | Ref. | 2 | 3 (1.5, 1.5-1.6) | Ref. |
| Second quintile | 2.3 | 4.8 (2.1, 2.1-2.2) | 3.3 (1.5, 1.4-1.5) | 1.1 (1.0-1.1) | 2.8 | 6 (2.1, 2.1-2.2) | 7.5 (2.7, 2.7-2.7) | 1.1 (1.1-1.1) | 2.3 | 4.6 (2.1, 1.9-2.2) | 1.3 (1.2-1.4) |
| Third quintile | 1.7 | 4.2 (2.4, 2.3-2.5) | 3 (1.7, 1.6-1.8) | 1.3 (1.2-1.4) | 1.8 | 4.7 (2.6, 2.6-2.7) | 5.9 (3.3, 3.2-3.4) | 1.4 (1.4-1.5) | 2 | 4.1 (2.0, 1.9-2.1) | 1.4 (1.3-1.5) |
| Fourth quintile | 1.1 | 3.1 (2.8, 2.6-3.0) | 2.1 (1.9, 1.8-2.1) | 1.5 (1.4-1.6) | 1.5 | 4.1 (2.8, 2.7-2.9) | 4.9 (3.3, 3.2-3.4) | 1.5 (1.4-1.6) | 1.6 | 2.9 (1.8, 1.7-1.9) | 1.3 (1.2-1.4) |
| Fifth quintile | .49 | 2.3 (4.7, 4.3-5.2) | 1.4 (2.7, 2.5-3.1) | 1.9 (1.7-2.1) | 1.2 | 3.3 (2.8, 2.6-3.0) | 3.8 (3.2, 3.0-3.4) | 1.5 (1.4-1.6) | 1.8 | 3.8 (2.1, 2.0-2.2) | 1.7 (1.6-1.8) |

aIRR, adjusted IRR; eq. P, P-value testing equality of IRR across groups; IR, incidence rate; IRR, incidence rate ratio. IR and IRR values calculated using multiple imputation for ethnicity values, with other missing data excluded. With adjustment for all variables listed in table; The ‘overall’ aIRR value represents a comparison to the baseline year for a 25-34 year-old woman of white British/Irish ethnicity living in an area with IMD in the first quintile, for other demographic variables such as age the aIRR values represent how much greater is the change from baseline year in each group of the variable relative to the respective reference group.

**Table S3:** New Chlamydia diagnoses analysed using Poisson regression models

|  | CSA1 [Pre-OPSS: Aug 2014-Jul 2015] | | | | CSA2 [Pre-OPSS: 2017] | | | | CSA3 [PreOPSS: 2019] | | |
| --- | --- | --- | --- | --- | --- | --- | --- | --- | --- | --- | --- |
|  | **Pre-OPSS;** IR/100PY | **Pre-COVID;** IR/100PY (IRR vs col1, 95%CI) | **2022;** IR/100PY (IRR vs Pre-OPSS, 95%CI) | **2022**; aIRR* (95%CI) | **Pre-OPSS;** IR/100PY | **Pre-COVID;** IR/100PY (IRR vs col1, 95%CI) | **2022;** IR/100PY (IRR vs Pre-OPSS, 95%CI) | **2022**; aIRR (95%CI) | **Pre-OPSS;** IR/100PY | **2022;** IR/100PY (IRR vs Pre-OPSS, 95%CI) | **2022**; aIRR* (95%CI) |
| % OPSS | 0% | 45.1% | 49.6% |  | 0% | 31.9% | 77.5% |  | 0% | 65.7% |  |
| Overall | .18 | .52 (2.8, 2.7-3.0) | .39 (2.1, 2.0-2.3) | 1.9 (1.7-2.2) | .25 | .43 (1.7, 1.6-1.7) | .46 (1.8, 1.7-1.9) | 1.9 (1.7-2.1) | .2 | .46 (2.2, 2.1-2.4) | 1.8 (1.5-2.2) |
| Population grouping *[missing, eq. P]* | 0.2% | 0.7%, <0.001 | 0.5%, <0.001 |  | 0.3% | 0.1%, <0.001 | 0.0%, <0.001 |  | 0.0% | 0.0%, <0.001 |  |
| Women | .18 | .59 (3.2, 3.0-3.4) | .42 (2.2, 2.1-2.4) | Ref. | .22 | .38 (1.7, 1.6-1.8) | .37 (1.7, 1.6-1.8) | Ref. | .16 | .49 (3.1, 2.8-3.5) | Ref. |
| MSEW | .15 | .34 (2.3, 2.1-2.5) | .26 (1.7, 1.6-1.9) | 0.8 (0.7-0.9) | .21 | .29 (1.3, 1.3-1.4) | .26 (1.2, 1.2-1.3) | 0.7 (0.7-0.8) | .18 | .31 (1.7, 1.5-1.9) | 0.6 (0.5-0.7) |
| MSM | 1.4 | 4.4 (3.2, 2.8-3.8) | 4.1 (3.0, 2.6-3.6) | 1.4 (1.1-1.6) | 1.6 | 3.8 (2.4, 2.2-2.6) | 5.3 (3.4, 3.1-3.6) | 1.9 (1.8-2.1) | 2.2 | 3.6 (1.6, 1.3-2.0) | 0.5 (0.4-0.7) |
| Age *[missing, eq. P]* | 0.0% | 0.0%, <0.001 | 0.0%, <0.001 |  | 0.0% | 0.0%, 0.383 | 0.0%, <0.001 |  | 0.0% | 0.0%, <0.001 |  |
| 16-24 | .59 | 1.9 (3.1, 2.9-3.4) | 1.3 (2.2, 2.1-2.4) | 1.3 (1.1-1.4) | .72 | 1.2 (1.6, 1.5-1.7) | 1.2 (1.6, 1.5-1.7) | 0.8 (0.8-0.9) | .6 | 1.7 (2.9, 2.6-3.2) | 1.8 (1.5-2.1) |
| 25-34 | .34 | .8 (2.3, 2.1-2.5) | .61 (1.8, 1.6-2.0) | Ref. | .41 | .71 (1.7, 1.6-1.8) | .78 (1.9, 1.8-2.0) | Ref. | .4 | .64 (1.6, 1.4-1.8) | Ref. |
| 35-44 | .09 | .21 (2.4, 2.0-2.9) | .22 (2.6, 2.1-3.1) | 1.4 (1.2-1.8) | .15 | .24 (1.6, 1.5-1.8) | .3 (2.0, 1.8-2.3) | 1.1 (1.0-1.2) | .12 | .17 (1.4, 1.0-1.8) | 0.9 (0.6-1.2) |
| 45-54 | .03 | .09 (3.5, 2.5-5.0) | .07 (2.9, 2.0-4.1) | 1.6 (1.1-2.3) | .07 | .13 (1.8, 1.5-2.1) | .14 (1.9, 1.6-2.2) | 1.0 (0.8-1.2) | .04 | .06 (1.6, 1.0-2.6) | 1.0 (0.6-1.6) |
| 55-64 | .01 | .03 (4.4, 2.2-9.2) | .03 (4.7, 2.3-9.6) | 2.6 (1.3-5.4) | .03 | .05 (1.9, 1.4-2.6) | .06 (2.3, 1.7-3.0) | 1.2 (0.9-1.6) | .02 | .01 (0.9, 0.4-2.2) | 0.6 (0.2-1.4) |
| ≥65 | 0 | .01 (3.3, 0.9-12.1) | 0 (2.0, 0.5-8.0) | 1.1 (0.3-4.5) | 0 | .01 (2.4, 1.0-5.4) | .01 (2.6, 1.2-5.9) | 1.4 (0.6-3.1) | 0 | .01 (6.0, 0.7-49.8) | 3.8 (0.5-31.4) |
| Ethnicity *[missing, eq. P]* | 6.7% | 7.0%, <0.001 | 12.9%, <0.001 |  | 14.8% | 19.2%, <0.001 | 4.9%, <0.001 |  | 5.5% | 16.7%, 0.008 |  |
| White – British/Irish | .13 | .45 (3.5, 3.3-3.9) | .32 (2.5, 2.3-2.7) | Ref. | .2 | .39 (1.9, 1.8-2.1) | .45 (2.2, 2.1-2.4) | Ref. | .18 | .44 (2.4, 2.2-2.6) | Ref. |
| White – other | .23 | .52 (2.3, 1.8-2.9) | .38 (1.7, 1.3-2.2) | 0.7 (0.5-0.9) | .34 | .54 (1.6, 1.5-1.7) | .52 (1.5, 1.4-1.6) | 0.7 (0.6-0.8) | .24 | .31 (1.3, 0.8-1.9) | 0.5 (0.3-0.8) |
| Black African | .34 | .97 (2.8, 2.3-3.4) | .98 (2.9, 2.4-3.5) | 1.2 (0.9-1.4) | .39 | .6 (1.5, 1.4-1.7) | .73 (1.9, 1.7-2.1) | 0.8 (0.7-1.0) | .3 | .65 (2.2, 1.5-3.2) | 0.9 (0.6-1.4) |
| Black Carib. or other | .99 | 2 (2.0, 1.8-2.2) | 1.5 (1.5, 1.4-1.7) | 0.6 (0.5-0.7) | .77 | 1.2 (1.5, 1.4-1.7) | 1 (1.3, 1.2-1.5) | 0.6 (0.5-0.7) | .36 | .74 (2.1, 1.2-3.6) | 0.9 (0.5-1.5) |
| Asian | .07 | .19 (2.7, 2.3-3.2) | .15 (2.2, 1.8-2.6) | 0.9 (0.7-1.1) | .1 | .15 (1.5, 1.4-1.7) | .14 (1.3, 1.2-1.5) | 0.6 (0.5-0.7) | .18 | .26 (1.5, 1.1-2.1) | 0.6 (0.4-0.9) |
| Any other | .06 | .18 (3.0, 1.9-4.9) | .17 (2.9, 1.7-4.7) | 1.2 (0.7-1.9) | .12 | .23 (1.9, 1.6-2.4) | .47 (3.9, 3.2-4.8) | 1.8 (1.4-2.2) | .1 | .33 (3.3, 1.6-6.6) | 1.4 (0.7-2.8) |
| Mixed ethnicity | .75 | 2 (2.7, 2.3-3.1) | 1.4 (1.9, 1.6-2.2) | 0.8 (0.6-0.9) | .63 | 1.1 (1.8, 1.6-2.0) | 1.2 (1.9, 1.7-2.1) | 0.9 (0.8-1.0) | .92 | 1.9 (2.1, 1.6-2.6) | 0.9 (0.7-1.1) |
| IMD *[missing, eq. P]* | 0.0% | 0.0%, <0.001 | 0.0%, <0.001 |  | 0.0% | 0.0%, <0.001 | 0.5%, <0.001 |  | 0.0% | 0.1%, 0.003 |  |
| First quintile | .23 | .62 (2.7, 2.5-2.9) | .44 (1.9, 1.8-2.0) | Ref. | .37 | .56 (1.5, 1.4-1.6) | .59 (1.6, 1.5-1.7) | Ref. | .23 | .41 (1.8, 1.6-2.1) | Ref. |
| Second quintile | .22 | .57 (2.6, 2.3-2.9) | .46 (2.1, 1.9-2.3) | 1.1 (1.0-1.3) | .3 | .48 (1.6, 1.5-1.7) | .51 (1.7, 1.6-1.8) | 1.1 (1.0-1.2) | .25 | .62 (2.5, 2.1-2.9) | 1.3 (1.0-1.6) |
| Third quintile | .13 | .45 (3.4, 2.9-3.9) | .39 (2.9, 2.5-3.5) | 1.7 (1.4-2.0) | .16 | .34 (2.1, 1.9-2.3) | .37 (2.3, 2.1-2.5) | 1.5 (1.4-1.7) | .19 | .47 (2.4, 2.0-2.9) | 1.4 (1.1-1.7) |
| Fourth quintile | .07 | .27 (3.8, 2.8-5.0) | .23 (3.2, 2.4-4.2) | 1.7 (1.3-2.3) | .14 | .26 (1.8, 1.6-2.1) | .3 (2.1, 1.8-2.4) | 1.4 (1.2-1.6) | .12 | .29 (2.4, 1.9-3.0) | 1.5 (1.1-2.0) |
| Fifth quintile | .04 | .22 (6.3, 4.4-9.0) | .14 (4.0, 2.8-5.9) | 2.0 (1.4-2.8) | .11 | .2 (1.8, 1.5-2.2) | .21 (1.8, 1.5-2.2) | 1.3 (1.1-1.6) | .15 | .42 (2.8, 2.3-3.4) | 1.9 (1.5-2.4) |

aIRR, adjusted IRR; eq. P, P-value testing equality of IRR across groups; IR, incidence rate; IRR, incidence rate ratio. IR and IRR values calculated using multiple imputation for ethnicity values, with other missing data excluded. With adjustment for all variables listed in table; The ‘overall’ aIRR value represents a comparison to the baseline year for a 25-34 year-old woman of white British/Irish ethnicity living in an area with IMD in the first quintile, for other demographic variables such as age the aIRR values represent how much greater is the change from baseline year in each group of the variable relative to the respective reference group.

**Table S4:** New gonorrhoea diagnoses analysed using Poisson regression models

|  | CSA1 [Pre-OPSS: Aug 2014-Jul 2015] | | | | CSA2 [Pre-OPSS: 2017] | | | | CSA3 [PreOPSS: 2019] | | |
| --- | --- | --- | --- | --- | --- | --- | --- | --- | --- | --- | --- |
|  | **Pre-OPSS;** IR/100PY | **Pre-COVID;** IR/100PY (IRR vs col1, 95%CI) | **2022;** IR/100PY (IRR vs Pre-OPSS, 95%CI) | **2022**; aIRR* (95%CI) | **Pre-OPSS;** IR/100PY | **Pre-COVID;** IR/100PY (IRR vs col1, 95%CI) | **2022;** IR/100PY (IRR vs Pre-OPSS, 95%CI) | **2022**; aIRR (95%CI) | **Pre-OPSS;** IR/100PY | **2022;** IR/100PY (IRR vs Pre-OPSS, 95%CI) | **2022**; aIRR* (95%CI) |
| % OPSS | 0% | 29.7% | 32.8% |  | 0% | 25.3% | 66.7% |  | 0% | 36.2% |  |
| Overall | .07 | .21 (3.3, 3.0-3.5) | .21 (3.2, 3.0-3.5) | 2.6 (2.1-3.2) | .11 | .19 (1.7, 1.6-1.8) | .24 (2.2, 2.1-2.3) | 2.3 (1.9-2.8) | .09 | .21 (2.3, 2.1-2.6) | 1.8 (1.3-2.4) |
| Population grouping *[missing, eq. P]* | 0.6% | 1.3%, 0.084 | 1.0%, 0.255 |  | 0.4% | 0.2%, <0.001 | 0.1%, <0.001 |  | 0.0% | 0.1%, 0.078 |  |
| Women | .04 | .16 (3.6, 3.2-4.2) | .16 (3.5, 3.0-4.0) | Ref. | .04 | .07 (1.8, 1.6-2.0) | .08 (2.0, 1.8-2.2) | Ref. | .06 | .17 (2.7, 2.2-3.2) | Ref. |
| MSEW | .04 | .12 (3.3, 2.8-3.8) | .13 (3.3, 2.8-3.9) | 0.9 (0.8-1.2) | .07 | .08 (1.2, 1.0-1.3) | .07 (1.1, 1.0-1.2) | 0.6 (0.5-0.7) | .05 | .11 (1.9, 1.5-2.4) | 0.7 (0.5-1.0) |
| MSM | 1.9 | 5.4 (2.9, 2.5-3.3) | 5.5 (3.0, 2.6-3.4) | 0.8 (0.7-1.0) | 2.2 | 4.2 (2.0, 1.8-2.1) | 6.1 (2.8, 2.6-3.0) | 1.4 (1.2-1.6) | 2 | 4.5 (2.3, 1.9-2.7) | 0.8 (0.6-1.1) |
| Age *[missing, eq. P]* | 0.0% | 0.0%, <0.001 | 0.0%, <0.001 |  | 0.0% | 0.0%, 0.310 | 0.0%, <0.001 |  | 0.0% | 0.0%, 0.436 |  |
| 16-24 | .19 | .55 (2.9, 2.6-3.3) | .52 (2.8, 2.5-3.1) | 0.9 (0.8-1.1) | .2 | .35 (1.7, 1.6-1.9) | .37 (1.8, 1.6-2.0) | 0.7 (0.6-0.8) | .23 | .6 (2.6, 2.2-3.1) | 1.2 (1.0-1.6) |
| 25-34 | .14 | .41 (3.0, 2.6-3.5) | .4 (2.9, 2.6-3.4) | Ref. | .18 | .32 (1.8, 1.7-2.0) | .44 (2.5, 2.3-2.7) | Ref. | .19 | .4 (2.1, 1.7-2.6) | Ref. |
| 35-44 | .04 | .17 (4.6, 3.5-6.0) | .2 (5.5, 4.2-7.2) | 1.9 (1.4-2.5) | .1 | .17 (1.6, 1.4-1.8) | .24 (2.3, 2.1-2.6) | 0.9 (0.8-1.1) | .07 | .14 (1.9, 1.3-2.6) | 0.9 (0.6-1.3) |
| 45-54 | .01 | .09 (8.4, 5.1-13.9) | .07 (7.2, 4.4-12.0) | 2.5 (1.5-4.2) | .08 | .11 (1.5, 1.2-1.7) | .13 (1.6, 1.4-1.9) | 0.7 (0.5-0.8) | .03 | .07 (2.0, 1.2-3.4) | 1.0 (0.6-1.6) |
| 55-64 | .01 | .03 (4.6, 2.2-9.9) | .03 (5.6, 2.7-11.9) | 1.9 (0.9-4.1) | .03 | .04 (1.8, 1.3-2.4) | .07 (2.6, 1.9-3.6) | 1.1 (0.8-1.5) | .01 | .02 (2.0, 0.8-5.3) | 0.9 (0.3-2.6) |
| ≥65 | 0 | 0 (2.0, 0.6-6.6) | .01 (3.0, 1.0-9.3) | 1.0 (0.3-3.2) | .01 | .01 (1.4, 0.7-2.6) | .01 (1.4, 0.8-2.7) | 0.6 (0.3-1.1) | 0 | .01 (3.0, 0.6-14.9) | 1.4 (0.3-7.1) |
| Ethnicity *[missing, eq. P]* | 5.5% | 8.1%, 0.055 | 14.5%, <0.001 |  | 11.3% | 18.7%, 0.030 | 6.5%, <0.001 |  | 8.4% | 26.1%, 0.319 |  |
| White – British/Irish | .06 | .18 (3.0, 2.7-3.4) | .17 (2.9, 2.5-3.3) | Ref. | .1 | .19 (1.9, 1.7-2.1) | .28 (2.8, 2.5-3.1) | Ref. | .08 | .2 (2.5, 2.1-2.8) | Ref. |
| White – other | .08 | .24 (2.9, 1.9-4.3) | .21 (2.6, 1.7-3.9) | 0.9 (0.6-1.4) | .16 | .28 (1.7, 1.5-1.9) | .35 (2.1, 1.9-2.4) | 0.8 (0.7-0.9) | .1 | .23 (2.3, 1.3-4.1) | 0.9 (0.5-1.7) |
| Black African | .06 | .28 (5.1, 3.2-8.0) | .39 (7.1, 4.5-11.0) | 2.4 (1.5-3.9) | .12 | .18 (1.5, 1.2-1.9) | .2 (1.7, 1.4-2.1) | 0.6 (0.5-0.8) | .09 | .31 (3.6, 1.8-7.4) | 1.5 (0.7-3.0) |
| Black Carib. or other | .26 | .84 (3.2, 2.6-3.9) | .8 (3.1, 2.5-3.7) | 1.1 (0.8-1.3) | .32 | .43 (1.4, 1.2-1.6) | .38 (1.2, 1.0-1.4) | 0.4 (0.4-0.5) | .15 | .18 (1.2, 0.4-3.2) | 0.5 (0.2-1.3) |
| Asian | .02 | .1 (4.6, 3.4-6.1) | .11 (4.8, 3.6-6.4) | 1.7 (1.2-2.3) | .04 | .07 (1.6, 1.3-1.9) | .07 (1.6, 1.3-1.9) | 0.6 (0.5-0.7) | .08 | .14 (1.8, 1.1-3.0) | 0.7 (0.4-1.2) |
| Any other | .04 | .08 (2.2, 1.2-4.2) | .11 (3.0, 1.6-5.6) | 1.0 (0.5-2.0) | .06 | .1 (1.9, 1.4-2.5) | .21 (3.8, 2.9-5.0) | 1.4 (1.0-1.8) | .09 | .13 (1.5, 0.6-3.6) | 0.6 (0.3-1.5) |
| Mixed ethnicity | .26 | .85 (3.2, 2.5-4.1) | .77 (2.9, 2.3-3.7) | 1.0 (0.8-1.3) | .26 | .46 (1.8, 1.5-2.2) | .55 (2.1, 1.8-2.5) | 0.8 (0.6-0.9) | .45 | .83 (1.9, 1.3-2.7) | 0.8 (0.5-1.1) |
| IMD *[missing, eq. P]* | 0.0% | 0.0%, 0.160 | 0.0%, 0.004 |  | 0.0% | 0.0%, 0.002 | 0.3%, <0.001 |  | 0.0% | 0.3%, <0.001 |  |
| First quintile | .09 | .27 (3.2, 2.9-3.6) | .26 (3.0, 2.7-3.4) | Ref. | .17 | .26 (1.6, 1.4-1.7) | .3 (1.8, 1.6-2.0) | Ref. | .11 | .19 (1.7, 1.4-2.0) | Ref. |
| Second quintile | .08 | .22 (2.9, 2.4-3.5) | .22 (2.9, 2.4-3.5) | 1.0 (0.8-1.2) | .13 | .22 (1.7, 1.6-1.8) | .28 (2.2, 2.0-2.3) | 1.2 (1.1-1.4) | .11 | .32 (2.8, 2.2-3.7) | 1.7 (1.3-2.4) |
| Third quintile | .04 | .16 (4.4, 3.3-5.8) | .19 (5.2, 3.9-6.9) | 1.8 (1.3-2.4) | .07 | .15 (2.1, 1.8-2.4) | .2 (2.8, 2.5-3.2) | 1.6 (1.4-1.9) | .09 | .22 (2.5, 1.9-3.3) | 1.7 (1.2-2.3) |
| Fourth quintile | .03 | .12 (3.6, 2.3-5.6) | .13 (3.9, 2.6-6.0) | 1.4 (0.9-2.1) | .06 | .11 (1.9, 1.5-2.3) | .15 (2.5, 2.1-3.0) | 1.5 (1.2-1.9) | .06 | .12 (2.1, 1.5-3.0) | 1.3 (0.9-2.0) |
| Fifth quintile | .02 | .06 (4.1, 2.3-7.2) | .07 (4.2, 2.4-7.4) | 1.3 (0.8-2.3) | .06 | .07 (1.2, 0.9-1.6) | .1 (1.8, 1.4-2.4) | 1.2 (0.9-1.6) | .05 | .18 (3.8, 2.7-5.3) | 2.7 (1.8-3.9) |

aIRR, adjusted IRR; eq. P, P-value testing equality of IRR across groups; IR, incidence rate; IRR, incidence rate ratio. IR and IRR values calculated using multiple imputation for ethnicity values, with other missing data excluded. With adjustment for all variables listed in table; The ‘overall’ aIRR value represents a comparison to the baseline year for a 25-34 year-old woman of white British/Irish ethnicity living in an area with IMD in the first quintile, for other demographic variables such as age the aIRR values represent how much greater is the change from baseline year in each group of the variable relative to the respective reference group.

**Table S5:** New HIV diagnoses analysed using Poisson regression models

|  | CSA1 [Pre-OPSS: Aug 2014-Jul 2015] | | | | CSA2 [Pre-OPSS: 2017] | | | | CSA3 [PreOPSS: 2019] | |
| --- | --- | --- | --- | --- | --- | --- | --- | --- | --- | --- |
|  | **Pre-OPSS;** IR/100kPY | **Pre-COVID;** IR/100kPY (IRR vs col1, 95%CI) | **2022;** IR/100kPY (IRR vs Pre-OPSS, 95%CI) | **2022**; aIRR* (95%CI) | **Pre-OPSS;** IR/100kPY | **Pre-COVID;** IR/100kPY (IRR vs col1, 95%CI) | **2022;** IR/100kPY (IRR vs Pre-OPSS, 95%CI) | **2022**; aIRR* (95%CI) | **Pre-OPSS;** IR/100kPY | **2022;** IR/100kPY (IRR vs Pre-OPSS, 95%CI) |
| % OPSS | 0% | 7.1% | 11.8% |  | 0% | 11.4% | 24.0% |  | 0% | 38.5% |
| Overall | 4.8 | 4 (0.8, 0.5-1.2) | 1.6 (0.3, 0.2-0.6) | 0.3 (0.1-1.4) | 6.2 | 4.7 (0.8, 0.6-1.0) | 2.7 (0.4, 0.3-0.6) | 0.2 (0.0-0.8) | .66 | 2.9 (4.3, 1.2-15.2) |
| Population grouping *[missing, eq. P]* | 0.0% | 0.0%, 0.699 | 0.0%, 0.802 |  | 0.8% | 2.2%, 0.256 | 0.0%, 0.273 |  | 0.0% | 0.0%, 0.856 |
| Women | 2.4 | 1.6 (0.7, 0.3-1.6) | .54 (0.2, 0.1-0.8) | Ref. | 2.2 | 2.1 (1.0, 0.5-1.8) | .82 (0.4, 0.2-0.9) | Ref. | 0 | .86 |
| MSEW | 2.6 | 2.8 (1.1, 0.5-2.3) | 1 (0.4, 0.1-1.1) | 1.8 (0.4-9.2) | 3.6 | 1.8 (0.5, 0.3-0.9) | .93 (0.3, 0.1-0.6) | 0.6 (0.2-1.9) | .46 | .93 (2.0, 0.2-22.1) |
| MSM | 178 | 135 (0.8, 0.4-1.4) | 64 (0.4, 0.2-0.8) | 1.6 (0.4-6.8) | 126 | 103 (0.8, 0.6-1.2) | 66 (0.5, 0.3-0.8) | 1.4 (0.6-3.5) | 26 | 119 (4.5, 1.0-20.8) |
| Age *[missing, eq. P]* | 0.0% | 0.0%, 0.265 | 0.0%, 0.775 |  | 0.0% | 0.0%, 0.823 | 0.0%, 0.525 |  |  |  |
| 16-24 | 4.9 | 2.7 (0.6, 0.2-1.7) | .54 (0.1, 0.0-0.9) | 0.3 (0.0-2.8) | 5.3 | 5.7 (1.1, 0.5-2.2) | .71 (0.1, 0.0-0.6) | 0.2 (0.0-1.1) |  |  |
| 25-34 | 13 | 7.8 (0.6, 0.3-1.1) | 4.7 (0.4, 0.2-0.8) | Ref. | 8.9 | 6.3 (0.7, 0.4-1.1) | 5 (0.6, 0.3-0.9) | Ref. |  |  |
| 35-44 | 5 | 5 (1.0, 0.4-2.5) | 3.3 (0.7, 0.2-1.9) | 1.9 (0.5-6.7) | 7.8 | 6.3 (0.8, 0.5-1.4) | 4.4 (0.6, 0.3-1.0) | 1.0 (0.5-2.2) |  |  |
| 45-54 | 1.8 | 6 (3.3, 0.9-12.1) | .6 (0.3, 0.0-3.2) | 0.9 (0.1-10.1) | 7.8 | 5.4 (0.7, 0.4-1.3) | 2.7 (0.3, 0.2-0.8) | 0.6 (0.2-1.6) |  |  |
| 55-64 | 2.8 | 1.4 (0.5, 0.1-2.7) | 0 (0.0, 0.0-.) | 0.0 (0.0-.) | 2.3 | .91 (0.4, 0.1-2.1) | 0 (0.0, 0.0-.) | 0.0 (0.0-.) |  |  |
| ≥65 | .51 | .51 (1.0, 0.1-16.0) | 0 (0.0, 0.0-.) | 0.0 (0.0-.) | 1.3 | .42 (0.3, 0.0-3.2) | 0 (0.0, 0.0-.) | 0.0 (0.0-.) |  |  |
| Ethnicity *[missing, eq. P]* | 9.8% | 11.9%, 0.218 | 17.6%, 0.959 |  | 19.5% | 24.4%, 0.966 | 20.0%, 0.713 |  |  |  |
| White – British/Irish | 4.9 | 2 (0.4, 0.2-0.8) | 1.8 (0.4, 0.2-0.8) | Ref. | 4.2 | 3.9 (0.9, 0.5-1.8) | 1.2 (0.3, 0.1-0.7) | Ref. |  |  |
| White – other | 5.1 | 3.6 (0.6, 0.1-6.9) | 3.3 (0.6, 0.1-6.9) | 1.7 (0.1-21.6) | 9.9 | 8 (0.8, 0.5-1.4) | 4.7 (0.5, 0.2-0.9) | 1.6 (0.5-5.0) |  |  |
| Black African | 18 | 14 (0.7, 0.3-2.2) | 2.8 (0.1, 0.0-1.1) | 0.4 (0.0-3.6) | 15 | 9.9 (0.7, 0.3-1.4) | 5.3 (0.4, 0.1-0.9) | 1.2 (0.3-4.7) |  |  |
| Black Carib. or other | 9.6 | 20 (2.1, 0.6-7.0) | 4 (0.4, 0.1-2.3) | 1.1 (0.2-7.3) | 11 | 8.7 (0.8, 0.3-2.1) | 4 (0.3, 0.1-1.4) | 1.2 (0.2-6.3) |  |  |
| Asian | 2.1 | 2.9 (1.4, 0.5-4.5) | .22 (0.0, 0.0-.) | 0.0 (0.0-.) | 2.5 | 1.4 (0.5, 0.2-1.8) | 1.2 (0.5, 0.1-1.5) | 1.5 (0.3-7.2) |  |  |
| Any other | 2.6 | 8.2 (3.2, 0.3-30.5) | 2.6 (1.0, 0.1-16.0) | 2.7 (0.2-47.5) | 3.9 | 1.7 (0.4, 0.0-3.4) | 5 (1.3, 0.4-4.8) | 4.4 (0.9-22.2) |  |  |
| Mixed ethnicity | 2.3 | 3.5 (3059.4, 0.0-.) | .58 (0.0, 0.0-.) | 0.0 (0.0-.) | 13 | 8.2 (0.6, 0.2-1.9) | 4.3 (0.3, 0.1-1.3) | 1.1 (0.2-6.2) |  |  |
| IMD *[missing, eq. P]* | 0.0% | 0.0%, 0.547 | 0.0%, 0.811 |  | 0.0% | 0.0%, 0.085 | 0.0%, 0.252 |  |  |  |
| First quintile | 5.8 | 5.8 (1.0, 0.6-1.7) | 2.3 (0.4, 0.2-0.8) | Ref. | 12 | 5.3 (0.4, 0.2-0.7) | 2.6 (0.2, 0.1-0.4) | Ref. |  |  |
| Second quintile | 4.3 | 2.4 (0.6, 0.2-1.7) | .48 (0.1, 0.0-0.9) | 0.5 (0.1-2.4) | 6.9 | 6.3 (0.9, 0.6-1.4) | 3.6 (0.5, 0.3-0.8) | 2.1 (0.9-5.1) |  |  |
| Third quintile | 4.5 | 2.6 (0.6, 0.2-2.0) | 0 (0, 0-0) | 0.0 (0.0-.) | 3.8 | 3.1 (0.8, 0.4-1.7) | 2.2 (0.6, 0.3-1.3) | 2.2 (0.8-6.2) |  |  |
| Fourth quintile | 6.2 | 1.2 (0.2, 0.0-1.7) | 1.2 (0.2, 0.0-1.7) | 0.5 (0.1-4.9) | 1.9 | 1.1 (0.6, 0.1-2.5) | 1.1 (0.6, 0.1-2.5) | 1.5 (0.3-7.0) |  |  |
| Fifth quintile | 0 | 2.1 (NA) | 3.1 (NA) | NA | .78 | 3.9 (5.0, 0.6-42.8) | .78 (1.0, 0.1-16.0) | 4.1 (0.2-72.2) |  |  |

aIRR, adjusted IRR; eq. P, P-value testing equality of IRR across groups; IR, incidence rate; IRR, incidence rate ratio. IR and IRR values calculated using multiple imputation for ethnicity values, with other missing data excluded. With adjustment for all variables listed in table; The ‘overall’ aIRR value represents a comparison to the baseline year for a 25-34 year-old woman of white British/Irish ethnicity living in an area with IMD in the first quintile, for other demographic variables such as age the aIRR values represent how much greater is the change from baseline year in each group of the variable relative to the respective reference group.

**Table S6:** Proportion of new Chlamydia diagnoses receiving treatment, or with care recorded as transferred to clinic following OPSS testing, analysed using log-link generalised linear models for binary outcomes.

|  | CSA1 [Pre-OPSS: Aug 2014-Jul 2015] | | | CSA2 [Pre-OPSS: 2017] | | | CSA3 [PreOPSS: 2019] | |
| --- | --- | --- | --- | --- | --- | --- | --- | --- |
|  | **Pre-OPSS;** n/N (%) | **Pre-COVID;** n/N (%) (RR vs col1, 95%CI) | **2022;** n/N (%) (RR vs col1, 95%CI) | **Pre-OPSS;** n/N (%) | **Pre-COVID;** n/N (%) (RR vs col1, 95%CI) | **2022;** n/N (%) (RR vs col1, 95%CI) | **Pre-OPSS;** n/N (%) | **2022;** n/N (%) (RR vs col1, 95%CI) |
| % OPSS | 0% | 45.1% | 49.6% | 0% | 31.9% | 77.5% | 0% | 65.7% |
| Overall | 1703/1954 (87.2) | 3724/5526 (67.4) (0.77, 0.75-0.79) | 2679/4168 (64.3) (0.74, 0.72-0.76) | 3913/4776 (81.9) | 6568/8052 (81.6) (1.00, 0.98-1.01) | 7870/8615 (91.4) (1.11, 1.10-1.13) | 820/933 (87.9) | 2026/2090 (96.9) (1.10, 1.08-1.13) |
| Population grouping *[missing, eq. P]* | 0.2% | 0.7%, 0.002 | 0.5%, <0.001 | 0.3% | 0.1%, 0.012 | 0.0%, <0.001 | 0.0% | 0.0%, 0.021 |
| Women | 853/1018 (83.8) | 2018/3225 (62.6) (0.75, 0.72-0.78) | 1401/2289 (61.2) (0.73, 0.70-0.76) | 1690/2127 (79.5) | 2965/3642 (81.4) (1.02, 1.00-1.05) | 3358/3634 (92.4) (1.16, 1.14-1.19) | 311/369 (84.3) | 1112/1143 (97.3) (1.15, 1.10-1.21) |
| MSEW | 679/745 (91.1) | 1251/1682 (74.4) (0.82, 0.79-0.85) | 822/1298 (63.3) (0.69, 0.66-0.73) | 1557/1838 (84.7) | 2012/2455 (82.0) (0.97, 0.94-0.99) | 2062/2250 (91.6) (1.08, 1.06-1.11) | 358/395 (90.6) | 651/671 (97.0) (1.07, 1.03-1.11) |
| MSM | 171/191 (89.5) | 455/619 (73.5) (0.82, 0.77-0.88) | 456/581 (78.5) (0.88, 0.82-0.94) | 666/811 (82.1) | 1591/1955 (81.4) (0.99, 0.95-1.03) | 2450/2731 (89.7) (1.09, 1.05-1.13) | 151/169 (89.3) | 263/276 (95.3) (1.07, 1.01-1.13) |

eq. P, P-value testing equality of RR across groups; RR, relative risk. n/N (%) values reported as observed, but RR values based on multiple imputation. *Results shown as % rather than n/N (%), based on average over multiple imputations. †statistical estimation of RR could not be performed across all imputations.

**Table S7:** Proportion of new gonorrhoea diagnoses receiving treatment , or with care recorded as transferred to clinic following OPSS testing, analysed using log-link generalised linear models for binary outcomes.

|  | CSA1 [Pre-OPSS: Aug 2014-Jul 2015] | | | CSA2 [Pre-OPSS: 2017] | | | CSA3 [PreOPSS: 2019] | |
| --- | --- | --- | --- | --- | --- | --- | --- | --- |
|  | **Pre-OPSS;** n/N (%) | **Pre-COVID;** n/N (%) (RR vs col1, 95%CI) | **2022;** n/N (%) (RR vs col1, 95%CI) | **Pre-OPSS;** n/N (%) | **Pre-COVID;** n/N (%) (RR vs col1, 95%CI) | **2022;** n/N (%) (RR vs col1, 95%CI) | **Pre-OPSS;** n/N (%) | **2022;** n/N (%) (RR vs col1, 95%CI) |
| % OPSS | 0% | 29.7% | 32.8% | 0% | 25.3% | 66.7% | 0% | 36.2% |
| Overall | 624/701 (89.0) | 1803/2286 (78.9) (0.89, 0.86-0.92) | 1760/2264 (77.7) (0.87, 0.84-0.90) | 1781/2097 (84.9) | 2874/3576 (80.4) (0.95, 0.92-0.97) | 4097/4553 (90.0) (1.06, 1.04-1.08) | 407/419 (97.1) | 940/972 (96.7) (0.99, 0.97-1.01) |
| Population grouping *[missing, eq. P]* | 0.6% | 1.3%, 0.148 | 1.0%, <0.001 | 0.4% | 0.2%, <0.001 | 0.1%, <0.001 | 0.0% | 0.1%, 0.070 |
| Women | 220/247 (89.1) | 679/899 (75.5) (0.85, 0.80-0.90) | 609/860 (70.8) (0.80, 0.75-0.85) | 331/396 (83.6) | 558/718 (77.7) (0.93, 0.88-0.99) | 707/789 (89.6) (1.07, 1.02-1.13) | 144/151 (95.4) | 390/405 (96.3) (1.01, 0.97-1.05) |
| MSEW | 175/191 (91.6) | 522/623 (83.8) (0.91, 0.87-0.97) | 511/628 (81.4) (0.89, 0.84-0.94) | 524/588 (89.1) | 536/683 (78.5) (0.88, 0.84-0.92) | 554/637 (87.0) (0.98, 0.94-1.02) | 115/118 (97.5) | 225/227 (99.1) (1.02, 0.98-1.05) |
| MSM | 229/263 (87.1) | 602/764 (78.8) (0.90, 0.85-0.96) | 640/776 (82.5) (0.95, 0.90-1.00) | 926/1113 (83.2) | 1780/2175 (81.8) (0.98, 0.95-1.02) | 2836/3127 (90.7) (1.09, 1.06-1.12) | 148/150 (98.7) | 325/340 (95.6) (0.97, 0.94-1.00) |

eq. P, P-value testing equality of RR across groups; RR, relative risk. n/N (%) values reported as observed, but RR values based on multiple imputation. *Results shown as % rather than n/N (%), based on average over multiple imputations. †statistical estimation of RR could not be performed across all imputations.

**Table S8:** Time in days from appointment date (clinic testing) or sample return date (OPSS testing) for patients with successful chlamydia treatment, analysed using linear regression models. All sites have data available for in-clinic positive cases. CSA2 includes only those with postal treatment among OPSS testers, and CSA3 does not include OPSS testers.

|  | CSA1 [Pre-OPSS: Aug 2014-Jul 2015] | | | CSA2 [Pre-OPSS: 2017] | | | CSA3 [PreOPSS: 2019] | |
| --- | --- | --- | --- | --- | --- | --- | --- | --- |
|  | **Pre-OPSS;** mean±SD | **Pre-COVID;**  mean±SD (δ, 95%CI) | **2022;**  mean±SD (δ, 95%CI) | **Pre-OPSS;** n/N (%) | **Pre-COVID;**  mean±SD (δ, 95%CI) | **2022;**  mean±SD (δ, 95%CI) | **Pre-OPSS;**  mean±SD | **2022;**  mean±SD (δ, 95%CI) |
| % OPSS | 0% | 32.6% | 39.4% | 0% | 19.5% | 74.4% | 0% | 0.0% |
| Overall | 4.7±7.5 | 7.9±9.2 (3.20, 2.73-3.66) | 10.7±8.3 (6.02, 5.54-6.49) | 5.2±6.6 | 5.0±6.0 (-0.19, -0.45-0.08) | 3.6±3.9 (-1.62, -1.85--1.39) | 3.2±4.3 | 5.7±6.5 (2.56, 1.98-3.14) |
| Population  grouping *[missing, eq. P]* | 0.0% | 0.1%, 0.732 | 0.3%, 0.004 | 0.1% | 0.1%, <0.001 | 0.1%, <0.001 | 0.0% | 0.0%, 0.312 |
| Women | 5.8±7.9 | 8.9±9.2 (3.08, 2.42-3.75) | 11.1±8.0 (5.28, 4.60-5.95) | 6.8±6.8 | 5.5±5.8 (-1.25, -1.65--0.84) | 3.4±3.5 (-3.34, -3.69--2.98) | 3.6±4.7 | 5.4±6.5 (1.86, 0.93-2.80) |
| MSEW | 3.1±6.3 | 5.9±8.8 (2.82, 2.13-3.51) | 10.1±9.0 (6.98, 6.20-7.77) | 3.2±5.6 | 3.5±5.6 (0.34, -0.05-0.73) | 3.2±4.1 (-0.00, -0.35-0.35) | 2.1±3.4 | 4.9±6.1 (2.86, 1.99-3.73) |
| MSM | 5.2±8.5 | 8.7±9.1 (3.43, 1.89-4.98) | 10.5±7.8 (5.30, 3.80-6.80) | 5.8±6.7 | 6.2±6.7 (0.44, -0.20-1.09) | 4.2±4.2 (-1.65, -2.20--1.10) | 4.8±4.5 | 7.3±6.9 (2.43, 1.15-3.70) |
| Age *[missing, eq. P]* | 0.0% | 0.0%, 0.102 | 0.0%, 0.528 | 0.0% | 0.0%, 0.011 | 0.0%, 0.005 | 0.0% | 0.0%, <0.001 |
| 16-24 | 4.8±7.7 | 8.2±9.4 (3.42, 2.79-4.05) | 11.0±8.4 (6.19, 5.54-6.85) | 5.3±6.6 | 5.1±6.1 (-0.24, -0.66-0.17) | 3.5±3.9 (-1.86, -2.22--1.50) | 3.3±4.7 | 5.2±6.3 (1.88, 1.06-2.70) |
| 25-34 | 4.6±7.4 | 6.9±8.5 (2.35, 1.56-3.14) | 10.6±8.2 (5.99, 5.15-6.83) | 5.1±6.8 | 4.6±5.7 (-0.56, -0.98--0.13) | 3.4±3.6 (-1.77, -2.15--1.40) | 2.9±3.8 | 6.1±6.1 (3.17, 2.24-4.11) |
| 35-44 | 3.8±7.1 | 8.4±10.2 (4.60, 2.90-6.29) | 9.8±8.6 (5.93, 4.38-7.48) | 4.7±5.7 | 5.3±6.4 (0.64, -0.07-1.36) | 4.1±4.5 (-0.56, -1.18-0.06) | 3.5±3.3 | 6.5±7.8 (3.06, 0.87-5.26) |
| 45-54 | 5.2±6.7 | 7.4±8.6 (2.19, -0.78-5.16) | 8.7±6.1 (3.49, 0.67-6.31) | 5.3±6.1 | 6.1±6.2 (0.82, -0.34-1.98) | 4.0±3.6 (-1.28, -2.29--0.28) | 2.7±3.6 | 8.0±8.3 (5.33, 2.29-8.37) |
| 55-64 | 3.4±5.2 | 7.9±8.0 (4.54, 0.23-8.84) | 10.9±7.1 (7.50, 3.40-11.61) | 5.5±6.5 | 6.5±7.4 (0.97, -1.39-3.32) | 4.8±5.1 (-0.75, -2.80-1.30) | 3.4±4.0 | 8.4±8.8 (5.03, -1.45-11.51) |
| ≥65 | 6.0±8.5 | 4.7±4.2 (-1.33, -10.20-7.53) | 9.6±2.6 (3.60, -5.39-12.59) | 3.8±3.3 | 7.2±6.3 (3.48, -0.86-7.82) | 4.6±3.3 (0.81, -2.38-3.99) | 10.0±. | 3.8±3.6 (-6.20, -9.06--3.34) |
| Ethnicity *[missing, eq. P]* | 6.5% | 6.4%, 0.089 | 13.6%, 0.102 | 13.7% | 22.5%, 0.014 | 5.5%, 0.013 | 4.9% | 48.9%, 0.589 |
| White – British/Irish | 4.4±7.1 | 8.0±8.9 (3.54, 2.72-4.35) | 11.1±8.2 (6.63, 5.75-7.52) | 5.1±6.4 | 4.3±5.2 (-0.78, -1.25--0.30) | 3.2±3.0 (-1.91, -2.36--1.46) | 3.0±4.3 | 5.8±6.7 (2.78, 2.05-3.51) |
| White – other | 4.2±5.7 | 9.4±10.3 (5.25, 3.00-7.49) | 11.9±9.0 (7.78, 5.37-10.19) | 5.4±6.4 | 5.4±6.5 (-0.02, -0.51-0.47) | 3.7±4.0 (-1.72, -2.19--1.24) | 4.2±4.0 | 5.8±6.3 (1.60, -2.77-5.97) |
| Black African | 4.3±7.9 | 8.3±9.6 (4.05, 2.33-5.76) | 10.3±8.6 (6.01, 4.25-7.78) | 5.3±6.8 | 5.2±6.2 (-0.07, -0.88-0.74) | 3.4±3.8 (-1.90, -2.67--1.13) | 3.4±4.0 | 6.2±6.1 (2.72, -0.56-6.00) |
| Black Carib. or other | 4.8±7.7 | 7.2±9.0 (2.41, 1.39-3.44) | 10.6±8.5 (5.80, 4.68-6.91) | 4.3±6.6 | 5.2±6.6 (0.87, 0.22-1.53) | 3.8±4.6 (-0.55, -1.21-0.12) | 3.4±4.4 | 4.7±5.7 (1.29, -2.36-4.95) |
| Asian | 4.5±7.2 | 8.2±9.1 (3.67, 2.11-5.23) | 10.3±8.3 (5.81, 4.16-7.45) | 5.4±6.7 | 5.3±5.8 (-0.07, -0.87-0.74) | 4.5±5.3 (-0.83, -1.60--0.07) | 3.9±4.8 | 5.8±6.0 (1.98, -0.31-4.26) |
| Any other | 7.7±8.7 | 8.9±10.5 (1.15, -3.44-5.75) | 9.6±8.4 (1.82, -2.84-6.49) | 5.7±7.0 | 4.8±6.0 (-0.94, -2.39-0.50) | 3.5±3.6 (-2.23, -3.49--0.98) | 5.1±5.4 | 3.7±4.7 (-1.45, -5.44-2.54) |
| Mixed ethnicity | 5.3±8.5 | 7.3±9.3 (1.98, 0.58-3.38) | 9.9±7.7 (4.59, 3.08-6.10) | 5.3±6.8 | 5.0±5.9 (-0.31, -1.10-0.48) | 3.5±4.1 (-1.77, -2.53--1.00) | 2.6±3.4 | 5.7±6.5 (3.03, 1.18-4.88) |
| IMD *[missing, eq. P]* | 0.0% | 0.0%, 0.182 | 0.0%, 0.378 | 0.0% | 0.0%, 0.002 | 0.3%, 0.282 | 0.0% | 0.0%, 0.322 |
| First quintile | 4.4±7.5 | 7.8±9.3 (3.35, 2.76-3.95) | 10.5±8.4 (6.08, 5.46-6.71) | 5.2±6.6 | 5.1±6.4 (-0.05, -0.58-0.49) | 3.7±4.1 (-1.52, -1.98--1.06) | 3.0±4.3 | 5.9±7.0 (2.95, 1.90-4.01) |
| Second quintile | 5.3±7.9 | 7.6±8.7 (2.33, 1.32-3.33) | 10.9±8.5 (5.62, 4.60-6.65) | 5.1±6.5 | 5.3±6.3 (0.19, -0.21-0.58) | 3.6±4.0 (-1.56, -1.90--1.22) | 3.2±3.7 | 6.3±7.2 (3.13, 1.85-4.42) |
| Third quintile | 4.9±7.3 | 9.3±9.7 (4.42, 2.98-5.85) | 11.5±8.0 (6.61, 5.23-7.99) | 5.1±6.6 | 4.5±5.4 (-0.59, -1.22-0.05) | 3.5±3.8 (-1.54, -2.12--0.97) | 3.0±4.2 | 5.2±5.6 (2.19, 1.04-3.34) |
| Fourth quintile | 5.0±6.8 | 7.6±8.8 (2.56, 0.14-4.97) | 9.3±7.0 (4.27, 1.95-6.58) | 5.9±6.9 | 4.2±5.0 (-1.78, -2.70--0.86) | 3.4±3.0 (-2.51, -3.34--1.68) | 3.8±4.3 | 5.0±5.2 (1.23, -0.32-2.77) |
| Fifth quintile | 4.0±4.5 | 7.0±8.3 (2.99, 0.87-5.12) | 11.0±7.9 (7.01, 4.67-9.36) | 4.5±6.2 | 4.5±4.4 (-0.01, -1.34-1.32) | 3.2±3.8 (-1.31, -2.61--0.02) | 3.4±5.0 | 5.6±6.2 (2.15, 0.53-3.77) |

eq. P, P-value testing equality of RR across groups; δ, mean difference. †statistical estimation of RR could not be performed across all imputations.

**Table S9:** Proportion of new gonorrhoea diagnoses with confirmed treatment that have a recorded test of cure 2-6 weeks after treatment date, among those testing in clinics, analysed using log-link generalised linear models for binary outcomes.

|  | CSA1 [Pre-OPSS: Aug 2014-Jul 2015] | | | CSA2 [Pre-OPSS: 2017] | | | CSA3 [PreOPSS: 2019] | |
| --- | --- | --- | --- | --- | --- | --- | --- | --- |
|  | **Pre-OPSS;** n/N (%) | **Pre-COVID;** n/N (%) (RR vs col1, 95%CI) | **2022;** n/N (%) (RR vs col1, 95%CI) | **Pre-OPSS;** n/N (%) | **Pre-COVID;** n/N (%) (RR vs col1, 95%CI) | **2022;** n/N (%) (RR vs col1, 95%CI) | **Pre-OPSS;** n/N (%) | **2022;** n/N (%) (RR vs col1, 95%CI) |
| % OPSS | 0% | 0.0% | 0.0% | 0% | 0.0% | 0.0% | 0% | 0.0% |
| Overall | 261/624 (41.8) | 600/1353 (44.3) (1.06, 0.95-1.19) | 397/1273 (31.2) (0.75, 0.66-0.84) | 338/1781 (19.0) | 491/2009 (24.4) (0.95, 0.86-1.05) | 98/1243 (7.9) (0.33, 0.27-0.41) | 207/407 (50.9) | 169/590 (28.6) (0.56, 0.48-0.66) |
| Population grouping *[missing, eq. P]* | 0.3% | 0.4%, 0.827 | 0.2%, 0.512 | 0.2% | 0.2%, 0.681 | 0.2%, 0.126 | 0.0% | 0.2%, 0.803 |
| Women | 96/220 (43.6) | 199/428 (46.5) (1.07, 0.89-1.28) | 111/331 (33.5) (0.77, 0.62-0.95) | 55/331 (16.6) | 95/370 (25.7) (1.05, 0.82-1.34) | 22/175 (12.6) (0.51, 0.33-0.78) | 69/144 (47.9) | 51/203 (25.1) (0.52, 0.39-0.70) |
| MSEW | 53/175 (30.3) | 152/477 (31.9) (1.05, 0.81-1.36) | 93/459 (20.3) (0.67, 0.50-0.89) | 47/524 (9.0) | 68/472 (14.4) (0.91, 0.67-1.22) | 17/295 (5.8) (0.29, 0.17-0.47) | 53/115 (46.1) | 40/158 (25.3) (0.55, 0.39-0.77) |
| MSM | 112/229 (48.9) | 249/448 (55.6) (1.14, 0.97-1.34) | 193/483 (40.0) (0.82, 0.69-0.97) | 236/926 (25.5) | 328/1167 (28.1) (0.94, 0.84-1.06) | 59/773 (7.6) (0.32, 0.25-0.41) | 85/148 (57.4) | 78/229 (34.1) (0.59, 0.47-0.75) |

eq. P, P-value testing equality of RR across groups; RR, relative risk. n/N (%) values reported as observed, but RR values based on multiple imputation. *Results shown as % rather than n/N (%), based on average over multiple imputations. †statistical estimation of RR could not be performed across all imputations.

**Table S10:** Proportion of HIV screen-positive results with confirmatory testing recorded within 6 weeks, among those testing in clinics, analysed using log-link generalised linear models for binary outcomes.

|  | CSA1 [Pre-OPSS: Aug 2014-Jul 2015] | | | CSA2 [Pre-OPSS: 2017] | | | CSA3 [PreOPSS: 2019] | |
| --- | --- | --- | --- | --- | --- | --- | --- | --- |
|  | **Pre-OPSS;** n/N (%) | **Pre-COVID;** n/N (%) (RR vs col1, 95%CI) | **2022;** n/N (%) (RR vs col1, 95%CI) | **Pre-OPSS;** n/N (%) | **Pre-COVID;** n/N (%) (RR vs col1, 95%CI) | **2022;** n/N (%) (RR vs col1, 95%CI) | **Pre-OPSS;** n/N (%) | **2022;** n/N (%) (RR vs col1, 95%CI) |
| % OPSS | 0% | 0.0% | 0.0% | 0% | 0.0% | 0.0% | 0% | 0.0% |
| Overall | 36/90 (40.0) | 53/82 (64.6) (1.62, 1.20-2.18) | 35/91 (38.5) (0.96, 0.67-1.38) | 190/246 (77.2) | 189/265 (71.3) (0.92, 0.83-1.02) | 128/163 (78.5) (1.02, 0.91-1.13) | 2/10 (20.0) | 9/26 (34.6) (1.73, 0.44-6.79) |
| Population grouping *[missing, eq. P]* | 0.0% | 0.0%, 0.026 | 1.1%, 0.352 | 0.8% | 1.5%, 0.035 | 0.0%, 0.903 | 0.0% |  |
| Women | 10/34 (29.4) | 23/32 (71.9) (2.44, 1.40-4.27) | 17/46 (37.0) (1.26, 0.67-2.37) | 50/62 (80.6) | 55/72 (76.4) (0.95, 0.79-1.13) | 35/43 (81.4) (1.01, 0.84-1.22) | 0/1 (0.0) | 3/5 (60.0) |
| MSEW | 16/27 (59.3) | 16/28 (57.1) (0.96, 0.62-1.49) | 9/22 (40.9) (0.69, 0.38-1.25) | 53/73 (72.6) | 43/52 (82.7) (1.14, 0.95-1.37) | 33/43 (76.7) (1.06, 0.85-1.31) | 2/4 (50.0) | 1/3 (33.3) |
| MSM | 10/29 (34.5) | 14/22 (63.6) (1.85, 1.01-3.36) | 9/23 (39.1) (1.13, 0.55-2.32) | 87/111 (78.4) | 91/141 (64.5) (0.82, 0.70-0.97) | 60/77 (77.9) (0.99, 0.85-1.16) | 0/5 (0.0) | 5/18 (27.8) |

eq. P, P-value testing equality of RR across groups; RR, relative risk. n/N (%) values reported as observed, but RR values based on multiple imputation. *Results shown as % rather than n/N (%), based on average over multiple imputations. †statistical estimation of RR could not be performed across all imputations.

**Table S11:** Detailed information regarding sex, gender and sexuality (as available) for sexual health service users in each year included in statistical analyses. Sexual health service users included based on a record of at least one chlamydia test within each year. For CSA2 and CSA3, users of both clinic and OPSS services in any given year will be double-counted as we do not have linkage between datasets.

|  | CSA1 [Pre-OPSS: Aug 2014-Jul 2015] | | | CSA2 [Pre-OPSS: 2017] | | | CSA3 [PreOPSS: 2019] | |
| --- | --- | --- | --- | --- | --- | --- | --- | --- |
|  | **PreOPSS**; n (%) | **Pre-COVID**; n (%) | **2022**; n (%) | **PreOPSS**; n (%) | **Pre-COVID**; n (%) | **2022**; n (%) | **PreOPSS**; n (%) | **2022**; n (%) |
| Clinic testers |  |  |  |  |  |  |  |  |
| Heterosexual. cis-man | 193 (1.1) | 811 (3.1) | 2014 (12.2) | 19738 (32.3) | 18680 (30.3) | 6290 (23.8) | 3292 (39.4) | 1885 (34.7) |
| Bisexual cis-man | 40 (0.2) | 131 (0.5) | 310 (1.9) | 1206 (2.0) | 1022 (1.7) | 679 (2.6) | 208 (2.5) | 205 (3.8) |
| Homosexual cis-man | 1393 (7.9) | 2289 (8.8) | 2030 (12.3) | 5350 (8.8) | 6802 (11.0) | 4292 (16.3) | 837 (10.0) | 798 (14.7) |
| Cis-man (unk. sexual.) | 6285 (35.5) | 8369 (32.0) | 3991 (24.1) | 327 (0.5) | 550 (0.9) | 285 (1.1) | 37 (0.4) | 22 (0.4) |
| Heterosexual cis-woman | 274 (1.5) | 1269 (4.9) | 2517 (15.2) | 33063 (54.1) | 32866 (53.3) | 13676 (51.9) | 3710 (44.4) | 2234 (41.1) |
| Bisexual cis-woman | 17 (0.1) | 62 (0.2) | 195 (1.2) | 593 (1.0) | 749 (1.2) | 504 (1.9) | 182 (2.2) | 172 (3.2) |
| Homosexual cis-woman | 3 (0.0) | 13 (0.0) | 22 (0.1) | 157 (0.3) | 209 (0.3) | 89 (0.3) | 26 (0.3) | 24 (0.4) |
| Cis-woman (unk. sexual.) | 9480 (53.5) | 13119 (50.2) | 5367 (32.5) | 532 (0.9) | 598 (1.0) | 457 (1.7) | 41 (0.5) | 36 (0.7) |
| Trans man | 0 (0.0) | 0 (0.0) | 0 (0.0) | 4 (0.0) | 12 (0.0) | 12 (0.0) | 1 (0.0) | 14 (0.3) |
| Trans woman | 0 (0.0) | 0 (0.0) | 0 (0.0) | 2 (0.0) | 10 (0.0) | 13 (0.0) | 3 (0.0) | 15 (0.3) |
| Transgender (unk. sex.) | 0 (0.0) | 2 (0.0) | 0 (0.0) | 6 (0.0) | 5 (0.0) | 0 (0.0) | 13 (0.2) | 7 (0.1) |
| Transgender or non-binary | 4 (0.0) | 28 (0.1) | 47 (0.3) | 0 (0.0) | 0 (0.0) | 0 (0.0) | 0 (0.0) | 0 (0.0) |
| Non-binary | 0 (0.0) | 0 (0.0) | 9 (0.1) | 9 (0.0) | 29 (0.0) | 57 (0.2) | 3 (0.0) | 15 (0.3) |
| No info. | 15 (0.1) | 55 (0.2) | 25 (0.2) | 61 (0.1) | 48 (0.1) | 4 (0.0) | 0 (0.0) | 3 (0.1) |
| OPSS testers |  |  |  |  |  |  |  |  |
| Heterosexual cis-man |  | 127 (0.5) | 228 (1.0) |  | 12090 (27.8) | 24258 (27.4) |  | 3826 (28.0) |
| Bisexual cis-man |  | 45 (0.2) | 96 (0.4) |  | 1109 (2.5) | 2597 (2.9) |  | 300 (2.2) |
| Homosexual cis-man |  | 2411 (8.9) | 1372 (6.2) |  | 5610 (12.9) | 12769 (14.4) |  | 798 (5.8) |
| Cis-man (unk. sexual.) |  | 6036 (22.3) | 5727 (25.8) |  | 157 (0.4) | 378 (0.4) |  | 52 (0.4) |
| Heterosexual cis-woman |  | 423 (1.6) | 771 (3.5) |  | 21588 (49.6) | 41837 (47.3) |  | 7627 (55.8) |
| Bisexual cis-woman |  | 22 (0.1) | 70 (0.3) |  | 2101 (4.8) | 4097 (4.6) |  | 666 (4.9) |
| Homosexual cis-woman |  | 2 (0.0) | 7 (0.0) |  | 507 (1.2) | 1273 (1.4) |  | 186 (1.4) |
| Cis-woman (unk. sexual.) |  | 17766 (65.7) | 13744 (61.9) |  | 124 (0.3) | 237 (0.3) |  | 43 (0.3) |
| Trans man |  | 0 (0.0) | 0 (0.0) |  | 16 (0.0) | 64 (0.1) |  | 27 (0.2) |
| Trans woman |  | 0 (0.0) | 0 (0.0) |  | 27 (0.1) | 142 (0.2) |  | 17 (0.1) |
| Transgender (unk. sex.) |  | 0 (0.0) | 0 (0.0) |  | 22 (0.1) | 1 (0.0) |  | 0 (0.0) |
| Transgender or non-binary |  | 72 (0.3) | 99 (0.4) |  | 0 (0.0) | 0 (0.0) |  | 0 (0.0) |
| Non-binary |  | 0 (0.0) | 4 (0.0) |  | 129 (0.3) | 781 (0.9) |  | 115 (0.8) |
| No info. |  | 143 (0.5) | 74 (0.3) |  | 0 (0.0) | 0 (0.0) |  | 0 (0.0) |

**Figure S1**: Positivity of chlamydia (CT) testing per episode of care. Columns correspond to the three Case Study Areas (CSA1-3), and data for each outcome are also split into: women, men who have sex exclusively with women (MSEW) and men who have sex with men (MSM).


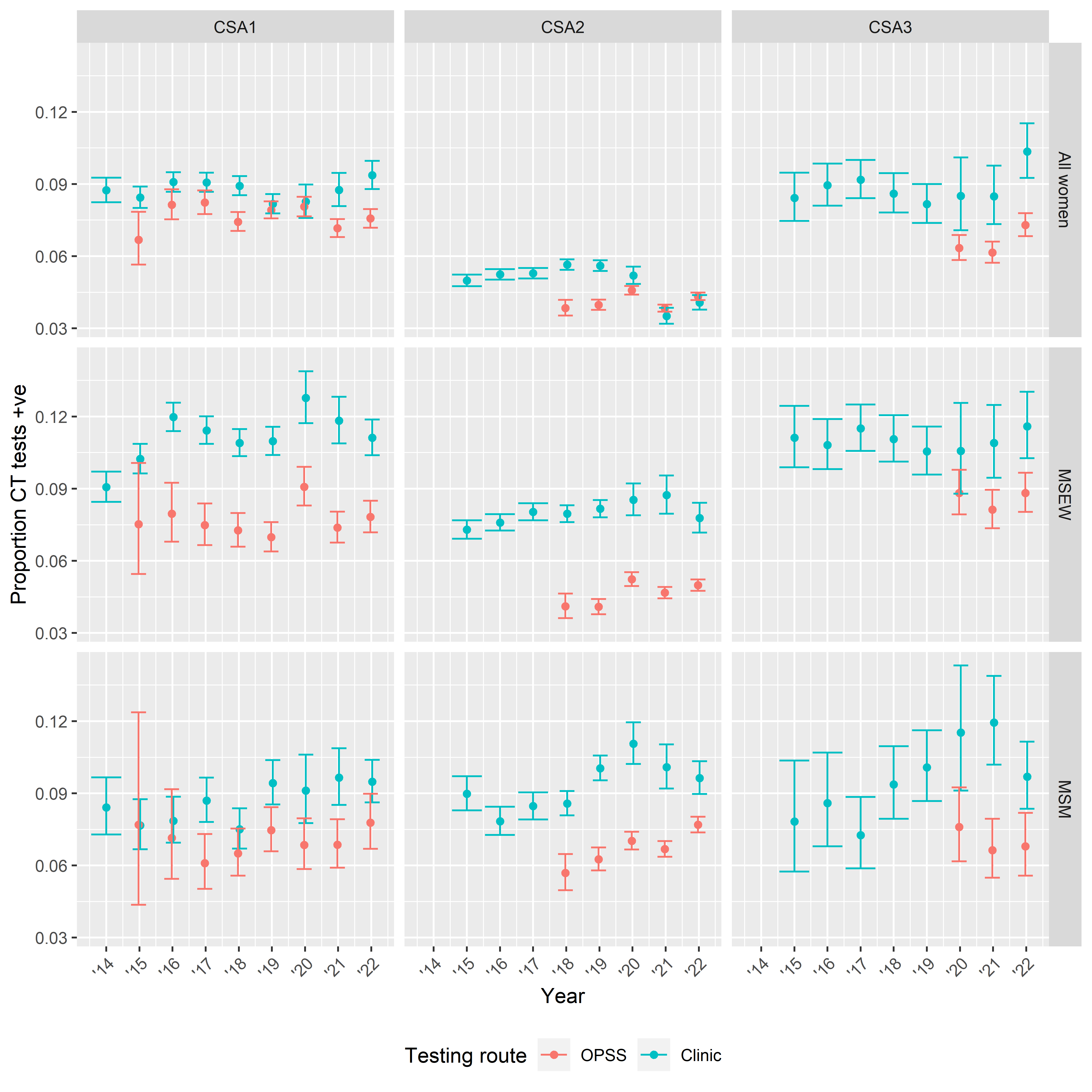


**Figure S2**: Positivity of gonorrhoea (NG) testing per episode of care. Columns correspond to the three Case Study Areas (CSA1-3), and data for each outcome are also split into: women, men who have sex exclusively with women (MSEW) and men who have sex with men (MSM).


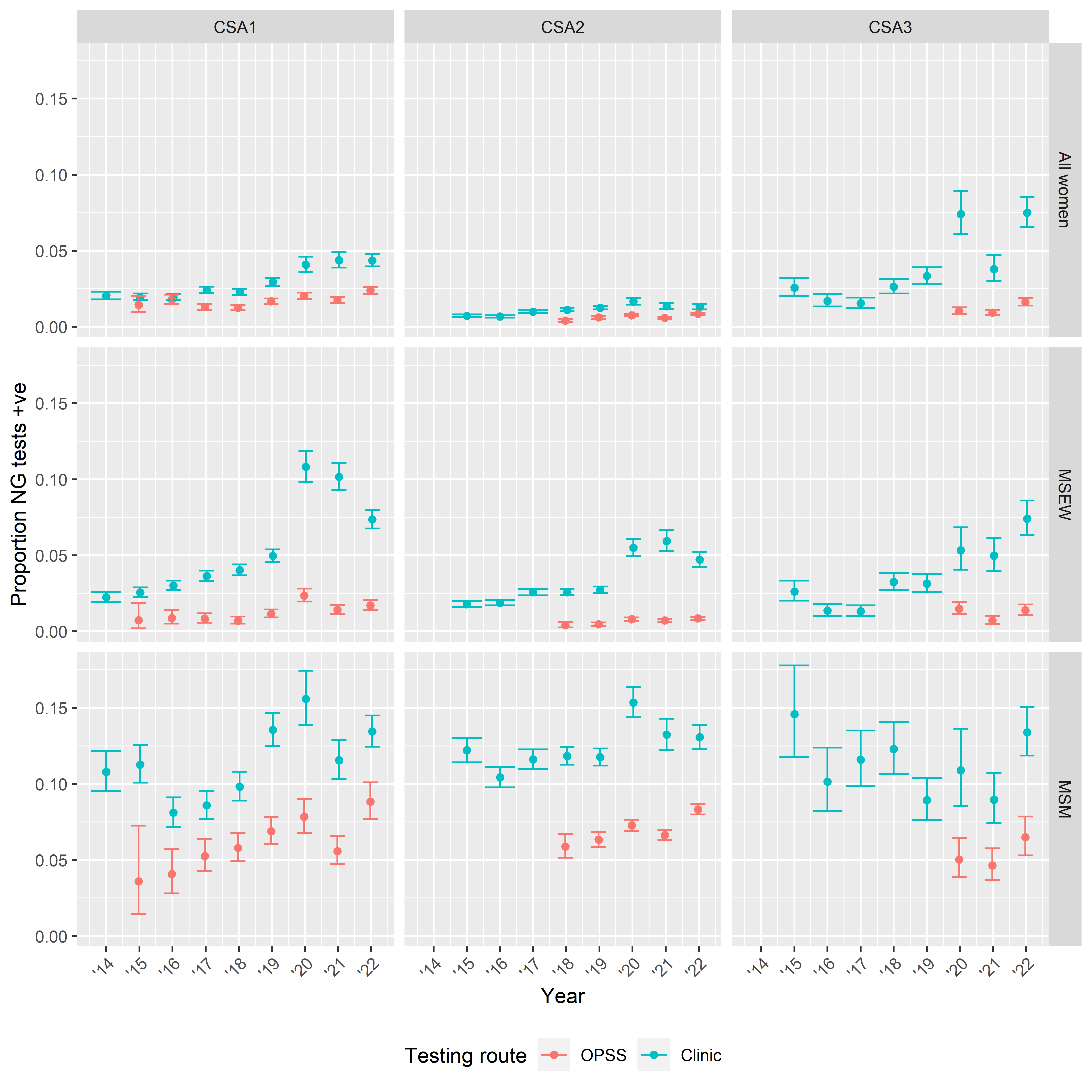


**Outcome definition details**

*Treatment definitions for chlamydia and gonorrhoea*

Appropriate treatment was defined as any record of prescription of doxycycline, azithromycin, erythromycin, ofloxacin or moxifloxacin for chlamydia, or ceftriaxone, ciprofloxacin, azithromycin or spectinomycin for gonorrhoea.

*Time-to-treatment outcomes for chlamydia*

Time to treatment for chlamydia was defined as time in days between appointment date for positive test and prescription date for clinic testers and between sample return date and prescription date for OPSS testers. If a prescription was issued within 7 days prior to apparent test date, then time to treatment was set to zero. Treatment records more than 6 weeks after test/sample return date were discarded. All sites have data available for in-clinic positive cases, but CSA2 includes only those with postal treatment among OPSS testers and CSA3 does not include OPSS testers.

*Confirmatory testing for HIV*

Any repeat antibody or viral RNA test within 6 weeks following a positive screening test was considered to constitute a confirmatory test. Viral RNA tests could be recorded on the same date as the initial screen-positive sample, but repeat antibody tests were required to be recorded at least one day after the initial positive sample (indicating that a separate sample has been provided for confirmation).

This outcome was only evaluated among those testing positive for HIV in clinic. The denominator for this outcome was anyone with a screen-positive (or equivocal) HIV antibody test, excluding anyone with a known prior diagnosis of HIV.

**Missing data and multiple imputation**

Initial inspection of the data revealed that of the main patient characteristics defined for the analysis, substantial missing data (i.e. >5%) was only present for ethnicity. For some sites there were also trends in the level of missing data for ethnicity over time, making it challenging to evaluate the impact of the introduction of OPSS according to ethnicity. To address this issue, we first considered multilevel multiple imputation (e.g. using the *jomo* package in R). However, this was not found to be feasible given the size and complexity of the datasets under investigation. We therefore conducted multiple imputation within Stata using only person-level covariables and then combined the results with the repeat episode of care observations for each person. Within the imputation process, data relating to individual test records were summarised for each individual as: total number of chlamydia tests, total number of chlamydia positive tests, total number of gonorrhoea positive tests, average date of testing activity.

Multiple imputation processes were run separately for each site, with the resulting set of imputed datasets used for analyses of all infections at each site, and included main covariables as in the statistical analyses (e.g. sex, gender and sexual behaviour group, age group, ethnicity, IMD quintile). We created 10 imputed datasets in each instance. Imputations will be stratified by clinic and OPSS data for CSA2 and CSA3, and for CSA1 the imputation process also included a person-level summary variable giving the proportion of OPSS testing episodes out of all of their testing activity.

Where a variable had missing data in less than 1% of the entire dataset, any affected individuals were excluded from the analyses.
